# Equivariant Neural Networks Reveal How Host–Guest Interactions Shape ^129^Xe NMR in Porous Liquids

**DOI:** 10.1021/acs.jpclett.5c02846

**Published:** 2025-11-11

**Authors:** Ouail Zakary, Perttu Lantto

**Affiliations:** † NMR Research Unit, Faculty of Science, University of Oulu, P.O. Box 3000, FI-90014 Oulu, Finland

## Abstract

Understanding host–guest interactions in porous liquids (PLs) formed from porous organic cages (POCs) is pivotal in tailoring their physicochemical properties, therefore providing an avenue for engineering new PLs with enhanced functionalities. In this work, we demonstrate, for the first time, the use of an accurate and efficient machine-learning-based approach for atomistic modeling of host–guest interactions in large-scale PLs. The approach uses *E*(3)-equivariant graph neural networks (EGNNs) to construct a machine learning interatomic potential and a nuclear magnetic resonance machine learning model. The former enables machine learning molecular dynamics (MLMD) simulations, while the latter computes the ^129^Xe isotropic chemical shift, δ_iso_, from MLMD snapshots. Applied to a PL composed of CC3-*R* POC in 4-(trifluoromethoxy)­benzyl alcohol (TBA) solvent loaded with high Xe concentration, this dual-model approach shows that host­(CC3)–guest­(Xe) interactions are best described by a three-site binding model comprising the CC3 intrinsic cavity, CC3 openings, and TBA solvent, with exchange events occurring between these sites. Good agreement between computed and experimental ^129^Xe δ_iso_ validates our approach, demonstrating EGNN-based simulations as transformative tools for advancing the understanding of PLs.

## Graphical abstract

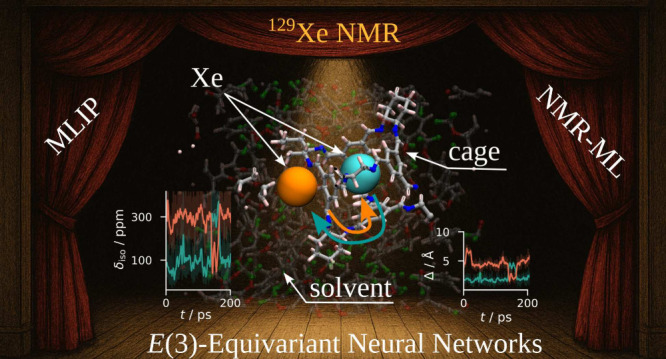

Porous materials (PMs) exhibit regions of empty space that enable selective adsorption and chemical transformation of guest molecules.^1^ This makes them essential for applications like adsorption, separation, catalysis, energy storage to production, and so on.^2,3^ While various classes of PMs are well-known, persistently porous liquids (PLs) remain rare, as most liquids lack permanent cavities.^4^ PLs represent a new category of PMs sharing the merits of both porous solids and flowing liquids.^4^ Significant advancements have been made in PL research since the concept was first put forward by O’Reilly et al. in 2007.^5^ These materials represent immense potential for enhanced gas selectivity, gas uptake, and lower energy requirement for regeneration.^6^ Such characteristics make PLs promising candidates for industrial applications like gas separation through continuous flow processes, offering energy-efficient methods to capture greenhouse gases like CO_2_ and CH_4_, as well as valuable noble gases (Ar, Kr, and Xe).^6–10^ PLs require a precise size selectivity and high adsorption capacity for effective gas isolation in commercial use. Recent development of PLs with cavities formed by porous organic cages (POCs) have shown potential to meet these challenges.^11–13^ For example, POCs like CC3 ^11^ and CC13 ^14^ have shown significantly enhanced uptake of CH_4_, CO_2_, Xe, N_2_, and SF_6_ compared to neat solvents, with gas uptake influenced by both the solvent and cage concentration.^15,16^ Interactions, including the binding, occupancies, and dynamics of the host (POC)–guest (gas) in equilibrium conditions, also influence gas uptake but remain not yet thoroughly understood. Exploring them is vital in engineering new PLs with an enhanced gas uptake. One method of studying these interactions is using xenon as a guest and analyzing its local environment with nuclear magnetic resonance (NMR) spectroscopy. The isotope ^129^Xe has a nuclear spin of 1/2, a relatively high gyromagnetic ratio, and highly sensitive chemical shift to its local environment. For instance, previous studies have shown that ^129^Xe NMR is an excellent probe for characterizing PMs.^17–26^ However, understanding the ^129^Xe isotropic chemical shift, δ_iso_, from only NMR spectra is challenging. The prediction of ^129^Xe spectroscopic responses by means of computational methods therefore plays an important role not only to assign spectral contributions but also to understand structural and dynamical origins of the experimental ^129^Xe NMR parameters. Computational methods such as molecular dynamics (MD) simulations help interpret NMR data. In MD simulations, atom movements are governed by forces derived as gradients of the potential energy surface (PES) of the system. Typically, the PES is accurately obtained using *ab initio* methods, such as density functional theory (DFT). While this combination of MD with DFT offers accurate interatomic forces, it remains limited to simulating only tens of picoseconds and a few hundred atoms, falling short of realistic time scales and sizes of these porous systems, ultimately leading to inaccurate interpretation of the NMR data. Semiempirical and classical PES models, although faster, lack accuracy.

Recently, machine learning interatomic potentials (MLIPs) have emerged as a promising solution to these limitations by learning accurate interatomic potentials from high-fidelity DFT data sets while maintaining computational efficiency.^27–42^ Indeed, MLIPs have demonstrated the ability to achieve near-quantum-mechanical accuracy at a fraction of the computational cost across a wide range of materials, including metal–organic frameworks,^43–53^ zeolites,^54–62^ liquid electrolytes,^63–67^ polymers,^68–71^ biomolecules,^72,73^ glasses and ceramics,^74–79^ as well as surfaces and interfaces.^80–83^ While early developments in the field of atomistic ML relied on descriptor-based ML models,^27,28^ more recent advances have introduced symmetry-aware graph neural network (GNN) approaches.^84–86^ These approaches use learned representations to capture the geometric characteristics of atomic environments while preserving fundamental symmetries, including rotational, translational, and permutational invariance. Invariant GNNs (IGNNs) achieve this by incorporating scalar features derived from interatomic distances,^87–89^ whereas equivariant GNNs (EGNNs) incorporate symmetry constraints directly into the model architecture, ensuring that internal representations and outputs transform consistently under symmetry operations.^90–100^ Both classes of models yield predictions that are physically consistent and invariant with respect to symmetry transformations. Moreover, both IGNNs and EGNNs can be used to predict NMR chemical shift tensors, with EGNNs generally demonstrating higher predictive accuracy than IGNNs.^96,101–103^ Despite these advances, PLs have not been modeled using ML-guided simulations, highlighting a promising avenue for the development of these materials.

Herein, we model for the first time host–guest interactions in PLs using EGNNs. Particularly, we investigate a PL composed of CC3-*R*, a POC with intrinsic cavity acting as the host, in 4-(trifluoromethoxy)­benzyl alcohol (TBA) and loaded with high Xe concentration, which we hereafter refer to as Xe@CC3@TBA. This system was chosen because of the availability of ^129^Xe NMR data,^25^ which provides a critical benchmark for assessing the accuracy of our EGNN-based simulations. Our approach employs the *E*(3)-EGNN architectures *Allegro* ^94^ and *MatTen* ^96,97^ to obtain an MLIP and an NMR machine learning (NMR-ML) model, respectively. The former enables machine learning molecular dynamics (MLMD) simulations, while the latter predicts the ^129^Xe magnetic shielding tensor, **σ**, from MLMD snapshots. This tensor is subsequently converted into a chemical shift tensor, **δ**, from which the isotropic chemical shift, δ_iso_, is computed and compared to the experimental data. The greater computational efficiency of MLMD simulations using *Allegro* ^94,104^ and NMR-ML predictions using *MatTen* ^96,97^ is well established in the literature, and our simulations are consistent with these findings. For example, a 1 ps DFT-level *ab initio* MD (AIMD) simulation of Xe in TBA solvent (Xe@TBA) took over 5 days using 1024 CPUs, whereas MLMD simulation took less than 3 min using one GPU. Similarly, ^129^Xe **σ** calculation performed on this system using DFT took over 60 h per MD snapshot, whereas it took less than one second using the NMR-ML model.

The MLIP was obtained following a one-cycle active learning method that consists of multiple steps illustrated in Figure S1 in the Supporting Information (SI). The first step consists of generating the initial Xe@CC3@TBA configuration, which corresponds to 1170 atoms with a Xe:CC3:TBA ratio of 2:1:50 (Figure 1 and Section I.1 in Supporting Information), followed by semiempirical MD simulations (SEMD-1), after which snapshots were sampled from the trajectories (Section I.2 in Supporting Information). Using PBE functional^105^ with D4 dispersion correction^106,107^ (PBE-D4), single-point DFT calculations were performed on the sampled snapshots to obtain properties, including the total energy, *E*, the atomic forces, f⃗ , and the stress tensor, ***s*** (Section I.3 in Supporting Information), which define the first data set (DFT-1). This data set was used to train *Allegro* architecture (Section I.4 in Supporting Information), resulting in the first-generation MLIP (MLIP-1). This interatomic potential was used to run the first round of MLMD simulations (MLMD-1), after which snapshots were sampled (Section I.5 in the Supporting Information). Single-point PBE-D4 calculations were performed on these snapshots to obtain *E*, f⃗ , and ***s***, which, along with their corresponding snapshots, were added to DFT-1 to build the second data set (DFT-2), consisting of 1001 snapshots. To verify the diversity of the conformational space of this data set, we perform dimensionality reduction analyses using principal component analysis (PCA)^108^ based on SOAP (smooth overlap atomic positions) descriptors^109^ (discussion in Section I.6 in SI and Figure S2). Furthermore, to gain a more detailed understanding of the local neighborhood structure and nonlinear relationships within this data set, we performed t-distributed stochastic neighbor embedding (t-SNE)^110^ analysis on individual atomic environments using both geometric (Cartesian) and chemical (SOAP) representations (discussion in Section I.6 in SI and Figure S2).

**Figure 1:**
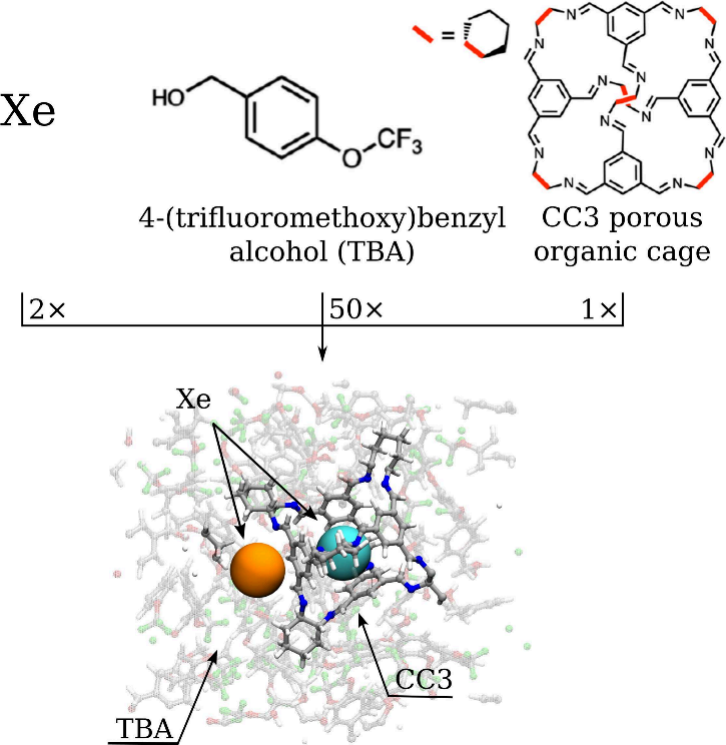
Schematic representation illustrating the construction of the simulation box for the porous liquid system, composed of CC3-R POC in 4-(trifluoromethoxy)­benzyl alcohol (TBA) and loaded with a high Xe concentration. The box corresponds to a Xe:CC3:TBA ratio of 2:1:50.

The DFT-2 data set was used to retrain *Allegro* architecture, resulting in the second-generation, final, MLIP (MLIP-2), with training and validation progress of the loss function, RMSE, and MAE illustrated in Figure S3. The accuracy of this model was evaluated by ML-predicted (DFT-computed) correlation plots from the test set of DFT-2 data set for *E*_DFT_ (*E*_ML_) (Figure 2a), ${\mathrm{f⃗}}_{\mathrm{DFT}}$ ( ${\mathrm{f⃗}}_{\mathrm{ML}}$ ) (Figure 2b), ***s***_DFT_ (***s***_ML_) (Figure 2c), force components (Figure S4), and stress tensor components (Figure S5). For all of these correlation plots, the linear least-squares regression yields a near-unity *R*-squared coefficient. The RMSEs (MAEs) are 1 (0.5) eV, 0.04 (0.02) eV.Å^–1^, and 0.25 (0.16) meV.Å^–3^ for *E*, f⃗ , and ***s***, respectively. These error magnitudes are well within acceptable ranges to run accurate MLMD simulations, particularly considering the chemical complexity of the Xe@CC3@TBA system.

**Figure 2:**
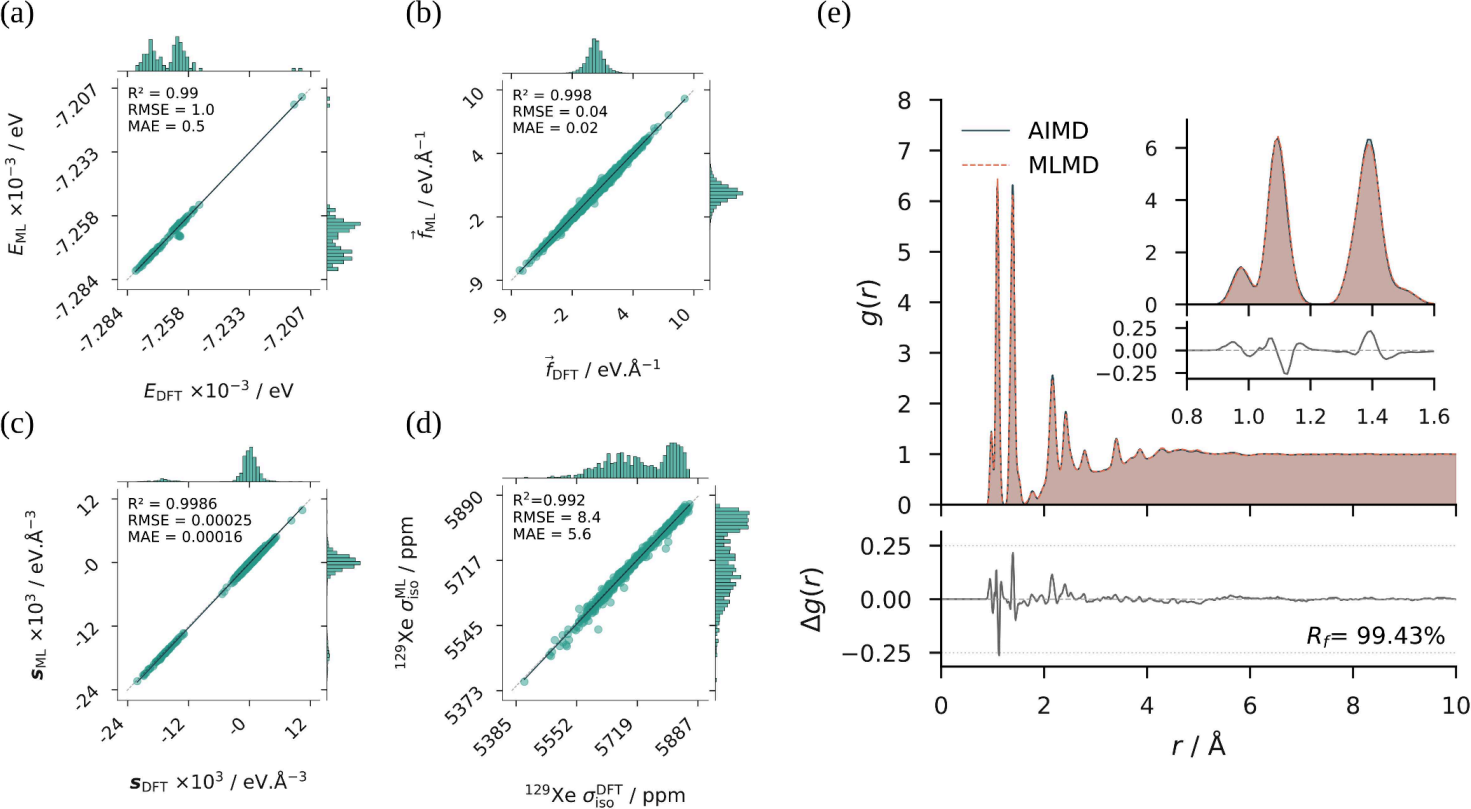
Correlation plots of the ML-predicted (DFT-computed) (a) *E*_ML_ (*E*_DFT_), (b) ${\mathrm{f⃗}}_{\mathrm{ML}}$ ( ${\mathrm{f⃗}}_{\mathrm{DFT}}$ ), and (c) ***s***_ML_ (***s***_DFT_), from the testing set of the DFT-2 data set, and (d) $\sigma_{\mathrm{iso}}^{\mathrm{ML}}$ ( $\sigma_{\mathrm{iso}}^{\mathrm{DFT}}$ ) from the testing set of the DFT-4 data set. The dashed line along the diagonal corresponds to *x* = *y*, while the solid line represents the linear least-squares regression fit. For each plot, the *R*-squared, RMSE, and MAE values are given in the upper left corner. Histograms along the axes show the projected distributions of ML-predicted and DFT-computed values from the testing data set. Total radial distribution functions are shown for all possible atom pairs, *g*(*r*), as a function of the interatomic distance, *r*, from AIMD (dark blue, e) and MLMD-2 (orange, e) simulation trajectories. The difference between the two curves, Δ*g*(*r*), is shown below in gray. The reliability factor, *R*_f_, quantifying the agreement between the two curves, is reported. An inset (top right, e) highlights the region 0.8Å ≤ *r* ≤ 1.6Å.

To assess the transferability of MLIP-2, we performed two tests (Figure S6) on a simpler system lacking CC3 cages and referred to here as Xe@TBA (Section II.1 in Supporting Information). The first test involved direct comparison between ML-predicted and DFT-computed *E*, f⃗ , and ***s*** from the third data set (DFT-3) that was constructed after SEMD simulations at RT and 600 K of Xe@TBA (SEMD-2, Section II.2 in Supporting Information), followed by single-point PBE-D4 calculations, while the second test compared trajectories from AIMD (Section II.3 in Supporting Information) with those from the second round of MLMD simulations (MLMD-2, Section II.4 in Supporting Information). The structural diversity of the Xe@TBA data sets (DFT-3 and AIMD) was assessed using PCA and t-SNE analyses (discussion in Section II.5 in SI and Figure S7). The ML-predicted and DFT-computed *E*, f⃗ , ***s*** at RT (Figures S8–S10) and 600 K (Figures S11–S13) show good agreement, with near-unity *R*-squared values and low RMSE and MAE values except for *E*, where a systematic shift is observed. The agreement between AIMD and MLMD-2 trajectories was quantified through computing *g*(*r*) curves (Section II.7 in Supporting Information) and comparing them, including those deduced from all possible atom pairs (Figure 2e) as well as from specific Xe–H, Xe–C, Xe–O, and Xe–F pairs (Figure S14). While the *g*(*r*) from all atom pairs show a good correspondence with a reliability factor of 99.4%, the pair-specific *g*(*r*) show less satisfactory agreement, which can be attributed to the limited phase-space sampling of the short AIMD trajectory (1 ps vs 10 ps for MLMD-2).

Following the accuracy and transferability of MLIP-2, we developed the NMR-ML model. This involves multiple steps illustrated in Figure S15, including performing the third sets of MLMD simulations (MLMD-3) at four temperatures (260, 280, 300, and 320 K) for Xe@CC3@TBA (Section III.1 in Supporting Information), sampling snapshots, computing ^129^Xe **σ**, then constructing the fourth data set (DFT-4) that includes the sampled snapshots with their corresponding ^129^Xe **σ** values. ^129^Xe **σ** was computed using DFT with the exact-two-component (X2C) scalar-relativistic Hamiltonian.^111^ Three sets of calculations were carried out, with Xe described in all cases using x2c-TZVPall-s basis set,^112^ while the remaining atoms (H, C, N, O, and F) were treated with different basis sets. These calculations used (*i*) PBE functional with x2c-SVPall basis set,^113^ (*ii*) PBE functional with x2c-TZVPall basis set, and (*iii*) hybrid BHandHLYP functional^114–116^ with x2c-SVPall basis set. All three calculations included a D4 dispersion correction (Section III.2 in Supporting Information). The diversity of this data set is illustrated by PCA and t-SNE analyses (discussion in Section III.3 in Supporting Information and Figure S16). This data set was then used to train *MatTen* architecture resulting in the NMR-ML model (Section III.4 in Supporting Information), with training and validation of the loss function, as well as validation of MAE illustrated in Figure S17. This model demonstrates strong predictive accuracy, with near-unity *R*-squared values for correlations between ML-predicted and DFT-calculated ^129^Xe σ_iso_ (Figure 2d) and **σ** components (Figure S18). The magnetic shielding tensor components show RMSE (MAE) values in the range of 9.7–10.5 (6.6–7.5) ppm for diagonal components and 4.8–5.4 (3.7–4) ppm for off-diagonal components. The ^129^Xe σ_iso_ RMSE (MAE) is 8.4 (5.6) ppm. These error rates are high; however, they remain within acceptable ranges for ^129^Xe.

Production MLMD simulations were performed on Xe@TBA (MLMD-4) and a 2 × 2 × 2 supercell of Xe@CC3@TBA (MLMD-5) as described in Figure S19 and Section IV.1 in SI. MLMD-4 was performed to test whether the NMR-ML is able to accurately describe ^129^Xe NMR in a neat liquid environment, while MLMD-5 was performed to investigate host–guest (CC3–Xe) dynamics and the corresponding Xe NMR signatures. For both MLMD trajectories, we computed ^129^Xe isotropic chemical shift, δ_iso_, and for MLMD-5 trajectory we computed, in addition, the displacement of Xe from the CC3 center of mass (Δ_Xe‑CM{CC3}_, Section IV.2 in Supporting Information). For MLMD-4, the evolution over time of ^129^Xe δ_iso_ (Figure S20) results in an average value of 148.8 ± 0.2 ppm, which agrees well with the experimental value, 147 ppm, in neat TBA liquid,^25^ highlighting the predictive capability of the NMR-ML model.

Generally, in the case where POCs are packed in the solid state, Xe atoms occupy two different environments with distinct ^129^Xe chemical shifts,^20^ including the intrinsic cage cavities, and the window cavity formed between two neighboring cages. As mentioned in previous work,^25^ discrete POCs in this PL are fully dissolved in the size-excluded solvent, eliminating window cavities and leaving only intrinsic cavities as primary binding sites. Furthermore, the TBA solvent needs to be taken into account as an additional environment, as Xe can dissolve in TBA. However, at high Xe loadings, i.e., Xe:CC3 molar ratio exceeding 1:1, another binding site emerges at the cage openings since each CC3 cavity accommodates only one Xe atom. Under these conditions, as shown by our MLMD-5 simulation, excess Xe atoms tend to cluster near the openings of already occupied cages, occasionally exchanging with Xe atoms inside the intrinsic cavities or migrating into the bulk TBA solvent. In this regard, and based on Δ_Xe‑CM{CC3}_, we defined three distinct binding sites: the CC3 cage (“*C*”, 0–4 Å), the CC3 cage openings or “*doors*” (“*D*”, 4–7 Å), and the TBA solvent (“*S*”, >7 Å) (Figure 3a). The evolution over time of both ^129^Xe δ_iso_ and Δ_Xe‑CM{CC3}_ (Figure 3b) for the CC3 cage highlighted in Figure 3c shows four distinct exchange events between two Xe atoms initially positioned at different sites (Xe@CC3@*t*_0_ and Xe@Door@*t*_0_), including back-and-forth exchanges between *C* and *D* sites, and one-directional exchange to an *S* site. These events were captured by sudden jumps in ^129^Xe δ_iso_ during its time-evolution, which show higher fluctuations than those of Δ_Xe‑CM{CC3}_ due to the high sensitivity of NMR chemical shifts to local environmental changes. Similar events were observed for the rest of the CC3 cages (Figure S21). The computed average ^129^Xe δ_iso_ values were 78.8 ± 0.3, 245.3 ± 0.5, and 201.2 ± 0.3 ppm for Xe at *C*, *D*, and *S* sites, respectively, reflecting the distinct chemical environments experienced by Xe at each location (Figure 3d,e). The overall average ^129^Xe δ_iso_ value is 147.2(±0.2) ppm, which shows a good agreement with the experimental value, 147.5 ppm, in the porous liquid with high-loading of Xe (HL PL, Figure 2 in ref 25), validating both the MLIP-2 dynamical and the NMR-ML chemical shift predictions. This agreement must, however, be considered within the context of the MLIP-2 and NMR-ML model RSMEs and MAEs as well as the systematic errors in the DFT levels of theory used for single-point and ^129^Xe **σ** tensor calculations. Nevertheless, this simulation remains quantitatively reliable, as the absolute differences in ^129^Xe δ_iso_ between the sites (166.5 ppm for *C*–*D*, 122.4 ppm for *C*–*S*, and 44.1 ppm for *D*–*S*) exceed the combined MAE, RMSE, and systematic errors.

**Figure 3:**
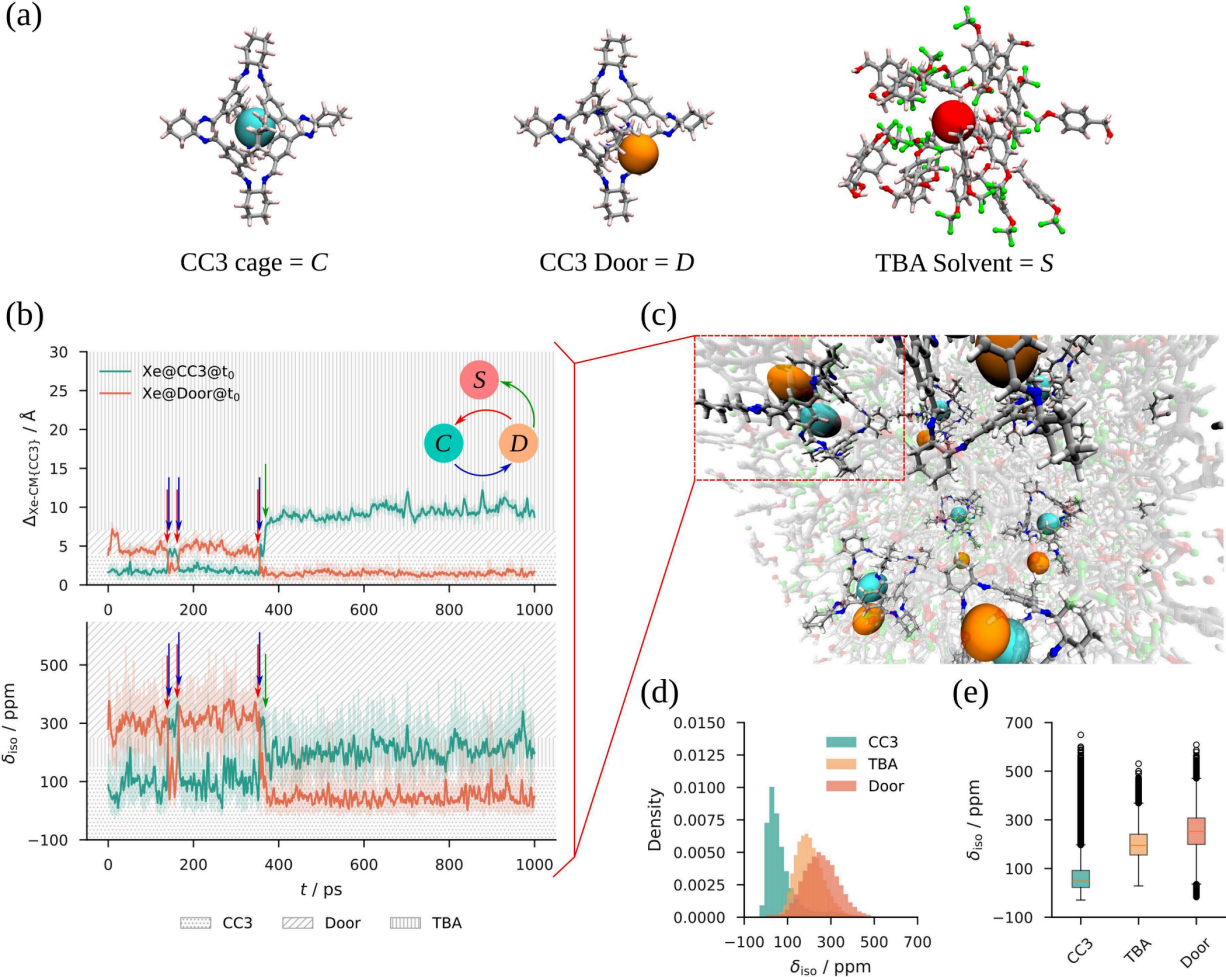
Schematic representation of the three binding sites, including the CC3 cage (left, a), the CC3 cage doors (middle, a), and the TBA solvent (right, a), labeled as “*C*”, “*D*”, and “*S*”, respectively. Evolution of Δ_Xe‑CM{CC3}_ and ^129^Xe δ_*iso*_ as a function of time (b) for the CC3 cage highlighted in red in the perspective view of Xe@CC3@TBA structure (c). In both plots, the *y*-axis is divided into three patterned regions where Xe is at *C* (dotted), *D* (///), and *S* (|||). Raw and filtered data are shown as semitransparent and opaque lines, respectively. Xenon atoms initially at *C* and *D* are labeled Xe@CC3@t_0_ (teal) and Xe@Door@t_0_ (orange), respectively, with *t*_0_ referring to their initial locations. Exchange events, where the two Xe atoms exchange sites, are indicated by colored arrows (top-right, b). Histograms showing the probability density distribution of ^129^Xe δ_iso_ for instances where Xe occupies *C* (teal), or *D* (orange), or *S* (light orange) sites, obtained from combined statistics across all CC3 cages in Xe@CC3@TBA MLMD-5 trajectory (d). Distribution of ^129^Xe δ_iso_ for the three binding sites, with the box plots show median (red line), interquartile range (IQR, box height), data range within 1.5 × IQR (whiskers), and outliers (individual points) (e).

This work demonstrates the first application of *E*(3)-EGNNs to model host–guest interactions in PLs at scale. Focusing on the Xe@CC3@TBA system, our dual-model approach, combining MLIP for molecular dynamics and NMR-ML for spectroscopic predictions, enables accurate atomistic modeling of this system. The good agreement between the computed and experimental ^129^Xe δ_iso_ validates our methodology and reveals a three-site binding model that governs host–guest interactions in this PL. Exchange events between the CC3 cage center of mass, CC3 cage openings, and TBA solvent sites are observed. This EGNN-based methodology is readily extensible to other host–guest environments and unlocks accurate large-scale MD simulations. This open the way to quantitative modeling of other relevant parameters, including exchange rates, residence times, and relaxation times, representing a significant step toward predictive modeling of complex porous liquid behavior across diverse chemical compositions and operating conditions.

## Supplementary Material



## Data Availability

All code created in this paper is publicly available in the ***GitHub*** repository: https://github.com/ozakary/data-Xe_at_CC3_at_TBA. The repository includes (*i*) input *DFTB+*, *VASP*, and *Turbomole* files for SEMD, DFT (and AIMD), and ^129^Xe **σ** simulations, respectively; (*ii*) *Python* scripts for the PCA and t-SNE analyses; (*iii*) data set preparation procedures and input *Allegro* and *MatTen* files for the MLIP and NMR-ML model, respectively; (*iv*) input *LAMMPS* files for the MLMD simulations; (*v*) code for predicting ^129^Xe **σ** using the NMR-ML model; and (*vi*) *Python* scripts for all figures used in the Letter and Supporting Information. The relevant data sets are publicly available in the ***Zenodo*** repository: 10.5281/zenodo.17105321. The repository includes (*i*) data sets used to train *Allegro* and *MatTen*, (*ii*) the MLIP-2 and NMR-ML model files, (*iii*) data set for the NMR-ML predictions, and (*iv*) data sets for all figures in the Letter and Supporting Information.

## Notes

Details related to (*i*) the steps used to obtain the MLIP, (*ii*) the steps used to evaluate the transferability of the MLIP, (*iii*) the steps used to obtain the NMR-ML model, and (*iv*) the MLMD production runs (PDF)
