# Supplementary material for: Equivariant Neural Networks Reveal How Host–Guest Interactions Shape 129Xe NMR in Porous Liquids
Source: J Phys Chem Lett. 2025 Nov 11;16(46):12095–103. doi: 10.1021/acs.jpclett.5c02846 (PMC12641476; doi:10.1021/acs.jpclett.5c02846)
Supplement: Supplementary file 1 [file jz5c02846_si_001.pdf]

Supporting Information for ”Equivariant Neural Networks Reveal How Host–Guest Interactions Shape ^{129}Xe NMR in Porous Liquids”

Ouail Zakary [1,*] and Perttu Lantto [1]

[1] *NMR Research Unit, Faculty of Science, University of Oulu, P. O. Box 3000, FI-90014 Oulu, Finland*

(Dated: October 13, 2025)

Content

I The Machine Learning Interatomic Potential S2
- I.1 The Initial Xe@CC3@TBA Configuration . . . S2
- I.2 Semi-Empirical Molecular Dynamics . . . S2
- I.3 Single-Point Calculations . . . S3
- I.4 The MLIP Neural Networks Architecture . . . S3
- I.5 Machine Learning Molecular Dynamics . . . S4
- I.6 PCA and t-SNE . . . S5
- I.7 Training and Validation Progress, and Tests of the MLIP . . . S7

II Transferability of the MLIP S10
- II.1 The Initial Xe@TBA Configuration . . . S10
- II.2 Semi-Empirical Molecular Dynamics . . . S10
- II.3 Single-point calculations and *Ab Initio* Molecular Dynamics . . . S10
- II.4 Machine Learning Molecular Dynamics . . . S11
- II.5 PCA and t-SNE . . . S11
- II.6 MLIP predictions: Xe@TBA at RT and 600 K . . . S12
- II.7 Radial Distribution Function . . . S16

III The Nuclear Magnetic Resonance Machine Learning Model S17
- III.1 Machine Learning Molecular Dynamics . . . S17
- III.2 ^{129}Xe $\boldsymbol{\sigma}$ Calculations . . . S17
- III.3 PCA and t-SNE . . . S18
- III.4 The NMR-ML Neural Network Architecture . . . S18
- III.5 Training and Validation Progress, and Tests of the NMR-ML Model . . . S20

IV Production Simulations S22
- IV.1 Machine Learning Molecular Dynamics . . . S22
- IV.2 Properties Simulations and Data Analysis . . . S22
- IV.3 The Production Machine Learning Molecular Dynamics Simulation . . . S23

References S25

* : corresponding author (E-mail: ouail.zakary@oulu.fi)

I. THE MACHINE LEARNING INTERATOMIC POTENTIAL

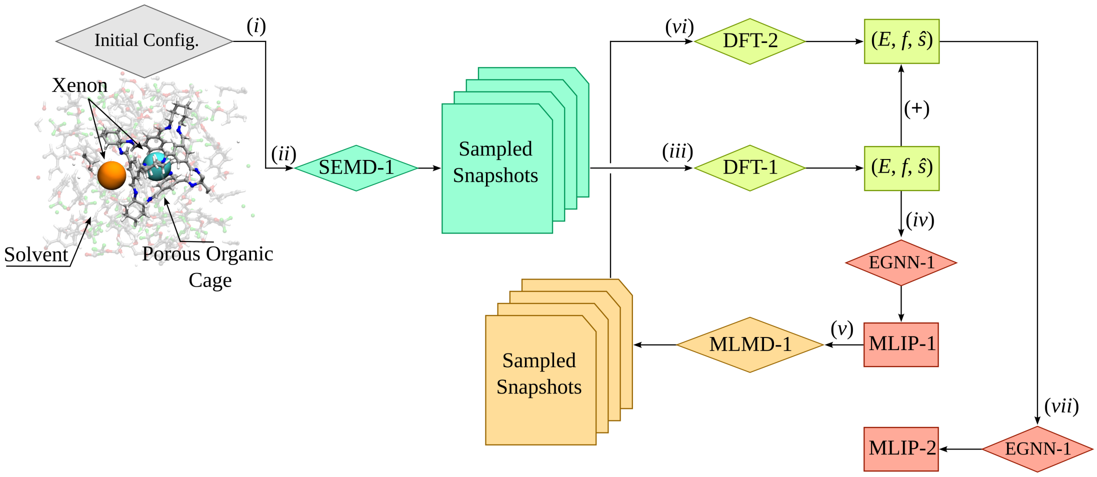

FIG. S1: Diagrammatic representation of the machine learning interatomic potential (MLIP) workflow, which include (i) building the initial configuration, (ii) performing semi-empirical molecular dynamics (SEMD) simulations and sample snapshots, (iii) carrying out the first round of single-point PBE-D4 calculations on the sampled snapshots to obtain total energies (E, eV), atomic forces ($\vec{f}$, eV.Å^{-1}), and stress tensors ($\boldsymbol{s}$, eV.Å^{-3}), which, together with the snapshots, constitute the first dataset (DFT-1), (iv) using DFT-1 dataset to train the local $E(3)$-equivariant graph neural network (EGNN-1) and obtain the first-generation MLIP (MLIP-1), (v) using MLIP-1 to perform the first round of machine learning molecular dynamics (MLMD-1) simulations and sample snapshots, (vi) performing the second round of single-point PBE-D4 calculations on the MLMD-1 snapshots to obtain E, $\vec{f}$, and $\boldsymbol{s}$, and merge these data with DFT-1 dataset to build the second dataset (DFT-2), and (vii) retraining EGNN-1 on DFT-2 dataset to obtain the second-generation MLIP (MLIP-2).

I.1. The Initial Xe@CC3@TBA Configuration

The initial Xe@CC3@TBA configuration was built using Packmol program (version 21.1.0)[1] through the AMSInput GUI (version 2024.107),[2] by placing the CC3 cage in a box and surrounding it with TBA solvent molecules. Since no experimental density is available for Xe@CC3@TBA, we adopted that of the neat TBA solvent at ambient conditions (1.33 g.mL^{-1}). To achieve this, the number of TBA molecules and the box dimensions were adjusted accordingly. The final system reached a density of 1.34 g.mL^{-1}, with a Xe:CC3:TBA ratio of 2:1:50. The 2:1 Xe-to-CC3 ratio was chosen to represent the high-loading case in ref([3]).

I.2. Semi-Empirical Molecular Dynamics

The first round of Semi-empirical MD simulations (SEMD-1) was performed on the initial Xe@CC3@TBA configuration, after which 281 snapshots (230 at 300 K and 51 at 600 K) were sampled. The simulations were carried out using the DFTB+ code (version 22.2),[4] with GFN2-xTB,[5,6] a three-bead Nosé–Hoover chain thermostat,[7] and the Velocity Verlet algorithm with a timestep of 0.5 fs. The system, comprising 1170 atoms in total, was modeled in a 23.9×23.9×23.9

Å^3 periodic box. The electronic structure was evaluated with Fermi–Dirac occupations at 300 K and 600 K under Γ-point sampling. The SEMD simulation was run for 67 ps, and snapshots were evenly sampled from 20 to 67 ps.

I.3. Single-Point Calculations

Single-point calculations were performed using VASP (version 6.4.1).[8,9] The electronic structure was computed through density functional theory (DFT) with periodic boundary conditions (PBC). The Perdew-Burke-Ernzerhof generalized gradient approximation functional (PBE)[10] was employed to obtain the ground state electronic structure. The London-dispersion correction D4[11,12] was used to account for van der Waals interactions present in the systems. The interactions between core and valence electrons were described using the projector augmented wave (PAW) method,[13] with core configurations of [He] for H, C, N, O, and F, and [Ar]$3d^{10}$ for Xe. The PAW potentials[14] used for H, C, N, O, F, and Xe are *H_GW_new*, *C_GW_new*, *N_GW_new*, *O_GW_new*, *F_GW_new*, and *Xe_sv_GW*, respectively. These potentials include one electron for H ($1s^1$), four electrons for C ($2s^2$ $2p^2$), five electrons for N ($2s^2$ $2p^3$), six electrons for O ($2s^2$ $2p^4$), seven electrons for F ($2s^2$ $2p^5$), and twenty-six electrons for Xe ($4s^2$ $4p^6$ $4d^{10}$ $5s^2$ $5p^6$). The wave functions were expanded on a plane-wave basis set with a 1000 eV kinetic energy cut-off and a Γ-only k-point mesh. The total energy was converged up to change below 10^{-6} eV.

I.4. The MLIP Neural Networks Architecture

We train *Allegro*,[15] a recently developed $E(3)$-EGNN.[16] Unlike many EGNNs that rely on message passing,[17] *Allegro* is strictly local, which enables efficient parallelization and supports simulations of very large systems.[15,18–20] The architecture and training pipeline were configured and optimized to balance model accuracy and computational efficiency, thereby avoiding memory overflow issues associated with overly complex models or large cutoff radii.

The *Allegro* architecture (version 0.2.0),[15] which requires *NequIP* (version 0.6.1),[21] operates with a maximum atomic interaction cutoff radius (r_{max}) of 6 Å, with the radial functions based on a trainable Bessel basis combined with an 8th-order polynomial envelope for smooth cutoff behavior at 8 Å. Spherical harmonics are used to handle symmetries with $l_{\max} = 2$ under full $O(3)$ symmetry. The network consists of four layers with an environmental embedding multiplicity of 16. The two-body latent multilayer perceptron (MLP) had three hidden layers with dimensions [32, 64, 128], using the "*SiLU*" nonlinearity and uniform initialization. The latent MLP consisted of one hidden layer of 128 nodes, also employing "*SiLU*" nonlinearity and uniform initialization, with residual connections enabled. The environmental embedding MLP was omitted, and the final MLP projecting latent features to edge energies used a single hidden layer of 128 units with uniform initialization and no nonlinearity.

Training was performed following a 8:1:1 dataset split, *i.e.*, 801, 100, and 100 snapshots for training, validation, and testing, respectively, with each set randomly sampled and shuffled after each epoch during the training. Data were loaded from an *ASE*-compatible (version 3.22.1)[22] "*.xyz*" file containing both atomic structures and their associated E, $\vec{f}$, and $\boldsymbol{s}$. Each atom was mapped to a chemical type (0, 1, 2, 3, 4, and 5 for O, C, H, F, Xe and N, respectively).

Optimization was conducted using the *Pytorch* (version 1.13)[23] *Adam* optimizer[24] with an initial learning rate of 0.002. A "*ReduceLROnPlateau*" learning rate scheduler was applied with a patience of 50 epochs and a decay factor of 0.5. Training was continued for a maximum of 10000 epochs or until early stopping criteria were met, with an early stopping learning rate floor of 10^{-5}. Batch size was set to 1, and an exponential moving average (EMA) of the weights was used with a decay rate of 0.99 and update-based averaging, to stabilize model convergence and improve generalization.

The loss function combined contributions from E, $\vec{f}$, and $\boldsymbol{s}$. Equal weighting was given to each property with total energies evaluated using the "*PerAtomMSELoss*" for improved scaling across systems of different sizes. Model performance was evaluated based on the validation loss, with detailed monitoring of the mean absolute error (MAE) and root mean square error (RMSE) of E, $\vec{f}$, and $\boldsymbol{s}$ (both per-system and per-atom normalized). The training was performed on a single NVIDIA Ampere A100 GPU with 40 GB of memory.

I.5. Machine Learning Molecular Dynamics

The first MLMD simulation round (MLMD-1) was carried out on LUMI supercomputer, using LAMMPS (version 2 Aug 2023 - Update 4)[25,26] with its multi-GPU implementation through Kokkos package (version 3.7.2)[27,28] for accelerated simulations. The simulation used 8× AMD MI250x GPUs (64 GB memory per GPU). The MLIP-1 was loaded through the "*pair_style allegro*" interface, using the deployed checkpoint file obtained after training *Allegro*. A binning neighbor list with a 1 Å skin distance was employed and updated every timestep. The system, comprising 1170 atoms in total, was modeled in a 23.9×23.9×23.9 Å^3 periodic box. The simulation was carried at 300 K, with velocities initialized according to this temperature, and thermal equilibration maintained using a Nosé–Hoover thermostat. The simulation began with a 0.1 ns equilibration phase, followed by a production run of 0.9 ns. The timestep used is 1 fs. System properties—including temperature and energies—were monitored every 100 steps, and snapshots were saved at the same frequency. 720 snapshots were sampled sequentially and added to the previous 281 snapshots from DFT-1 dataset, resulting in 1001 snapshots.

I.6. PCA and t-SNE

SOAP (Smooth Overlap of Atomic Positions)[29] descriptors were computed using the DScribe library (version 2.1.1),[30,31] with a cutoff radius of 5 Å, 8 radial basis functions, 6 angular basis functions, and a Gaussian width of 0.3 Å. Periodic boundary conditions were applied to accurately represent the bulk solution environment consistent with the original MD simulations. The SOAP descriptors were calculated for all atoms and subsequently averaged to obtain a single representative descriptor vector per snapshot. This approach yielded a dataset of 1001 and 1247 descriptor vectors for Xe@CC3@TBA and Xe@TBA, respectively, each containing 3330 features corresponding to the SOAP representation of the averaged atomic environments.

The principal component analysis (PCA)[32] was performed using *scikit-learn* (version 1.4.2)[33] without additional feature scaling, as SOAP descriptors are inherently normalized. The analysis retained two principal components to enable visualization of dataset diversity in a two-dimensional projection.

To gain more insight into the dataset diversity, t-distributed stochastic neighbor embedding (t-SNE) analysis[34] was used. This analysis examined individual atomic environments rather than structure-averaged properties, providing insight into the local chemical diversity present in the dataset. From the total of approximately 1.2 and 1.3 million atomic environments across the datasets, a representative subset of 3×10^4 neighborhoods was randomly selected to maintain computational feasibility while preserving statistical representativeness.

Two complementary representations were employed to capture different aspects of atomic environments. The Cartesian representation computed geometric features for each atom, including the 20 shortest interatomic distances arranged in ascending order and coordination numbers within 2, 3, and 4 Å radii. Distance vectors were zero-padded to ensure uniform dimensionality across all atomic environments. This approach provides a geometrically intuitive but rotationally variant description of local atomic structure.

The SOAP representation applied the same parameters used in PCA to individual atomic environments. This representation captures the chemical environment around each atom in a rotationally invariant manner, enabling direct comparison with the structure-averaged analysis while preserving information about local heterogeneity.

Both feature matrices were standardized using "*StandardScaler*" implemented in *scikit-learn* (version 1.4.2) to achieve zero mean and unit variance for optimal t-SNE performance. The t-SNE algorithm was implemented using *scikit-learn* (version 1.4.2) with the Barnes-Hut approximation[35] for computational efficiency. Key parameters included a perplexity of 30, learning rate of 200, maximum of 1000 iterations, and a fixed random state of 42 for reproducibility. The quality of the resulting embeddings was assessed using the final Kullback-Leibler divergence values,[36] with lower values indicating better preservation of local neighborhood structure in the two-dimensional projection.

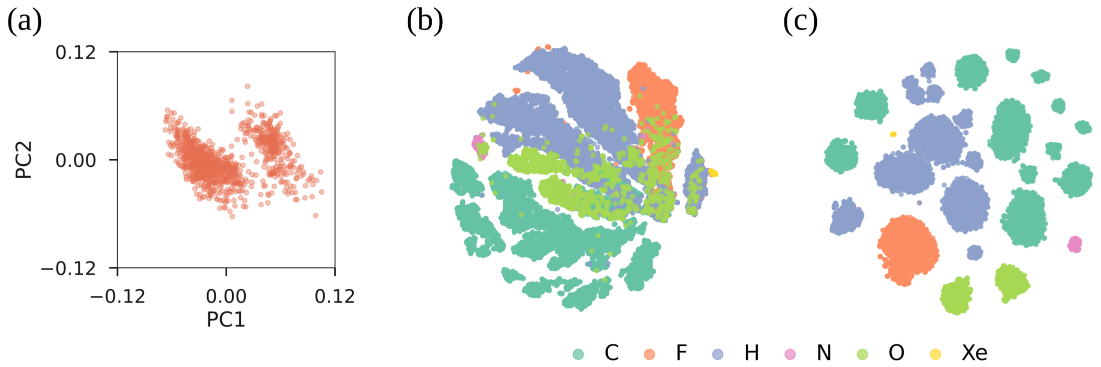

FIG. S2: PCA of DFT-2 dataset in the first two principal components (PC1, PC2) based on structure-averaged SOAP descriptors (a). t-SNE of $3{\times}10^4$ randomly sampled individual atomic neighborhoods from the DFT-2 datasets using Cartesian (b) and SOAP (c) representations, with points colored by atomic species.

The two first principle components capture 70.3% of the total variance (PC1: 53.3%, PC2: 16.9%), demonstrating a wide-range conformational space across both components with PC1 capturing the dominant mode of structural variations (**Figure S2a**). The analysis identified 6.5%, 1.2%, and 0.2% snapshots exceeding 2×, 2.5×, and 3× standard deviation from the mean, respectively. These snapshots, while uncommon, they contribute to the diversity of DFT-2 dataset.

Unlike PCA, which captures global linear variance, t-SNE preserves local topology and can reveal clustering patterns that may not be apparent in linear projections. The analysis of $3{\times}10^4$ randomly sampled individual atomic neighborhoods showed distinct clustering behavior for different atomic species, with the SOAP representation revealing more chemically meaningful groupings compared to the purely geometric Cartesian features. The t-SNE embedding based on the Cartesian representation does not form discrete or isolated clusters but rather continuous distributions, indicating that the local atomic environments sampled in the dataset vary gradually rather than abruptly (**Figure S2b**). Moreover, the well-separated clusters for different atom types from SOAP descriptors indicate that the dataset contains chemically diverse local environments (**Figure S2c**).

I.7. Training and Validation Progress, and Tests of the MLIP

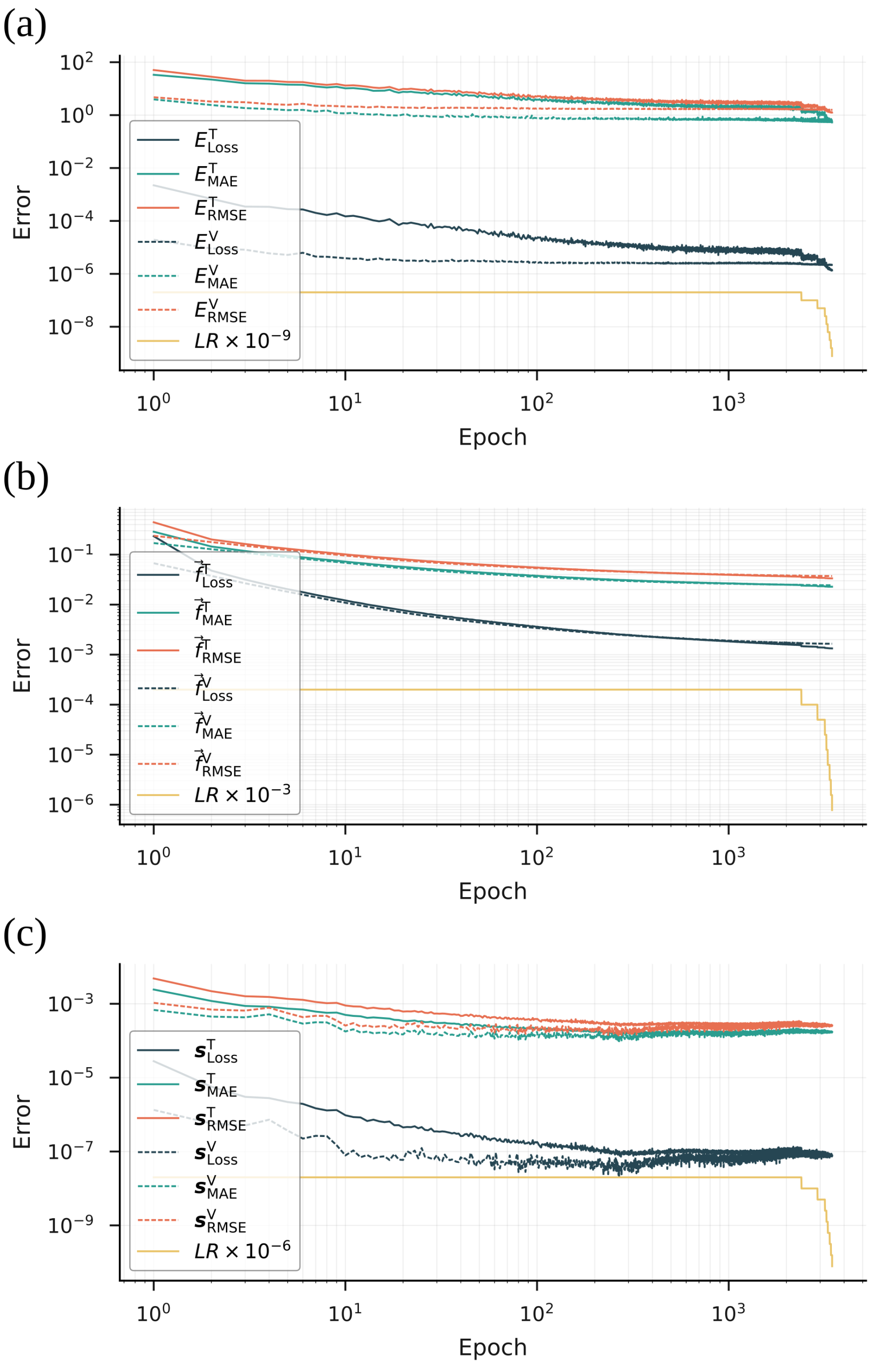

FIG. S3: Training and validation progress from *Allegro* for Xe@CC3@TBA. The figure illustrates the evolutions of the training (T) and validation (V) mean absolute error (MAE), root mean squared error (RMSE), and loss function for (a) E (E^{T}_{MAE}, E^{V}_{MAE}, E^{T}_{RMSE}, E^{V}_{RMSE}, E^{T}_{Loss}, and E^{V}_{Loss}, eV), (b) $\vec{f}$ ($\vec{f}^{T}_{MAE}$, $\vec{f}^{V}_{MAE}$, $\vec{f}^{T}_{RMSE}$, $\vec{f}^{V}_{RMSE}$, $\vec{f}^{T}_{Loss}$, and $\vec{f}^{V}_{Loss}$, eV.Å^{-1}), and (c) $\boldsymbol{s}$ ($\boldsymbol{s}^{T}_{MAE}$, $\boldsymbol{s}^{V}_{MAE}$, $\boldsymbol{s}^{T}_{RMSE}$, $\boldsymbol{s}^{V}_{RMSE}$, $\boldsymbol{s}^{T}_{Loss}$, and $\boldsymbol{s}^{V}_{Loss}$, eV.Å^{-3}). The learning rate (LR) is shown in yellow.

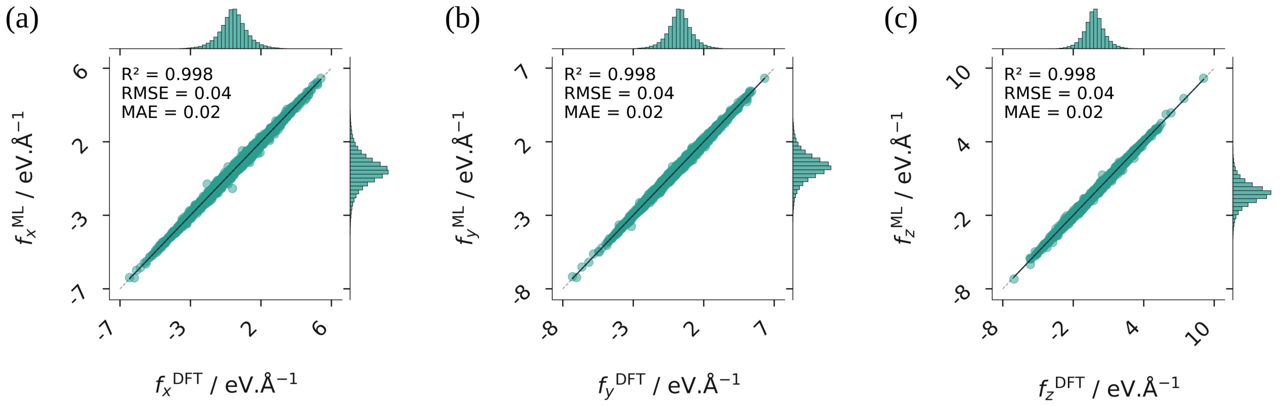

FIG. S4: Correlation plots of the ML-predicted (DFT-computed) atomic force components (a) f_x^{ML} (f_x^{DFT}), (b) f_y^{ML} (f_y^{DFT}), and (c) f_z^{ML} (f_z^{DFT}), from the testing dataset of Xe@CC3@TBA. The dashed line along the diagonal corresponds to $x = y$, while the solid line represents the linear least-squares regression fit. For each plot, the R-squared, RMSE, and MAE values are given in the upper left corner. Histograms along the axes show the projected distributions of ML-predicted and DFT-computed values from the testing dataset.

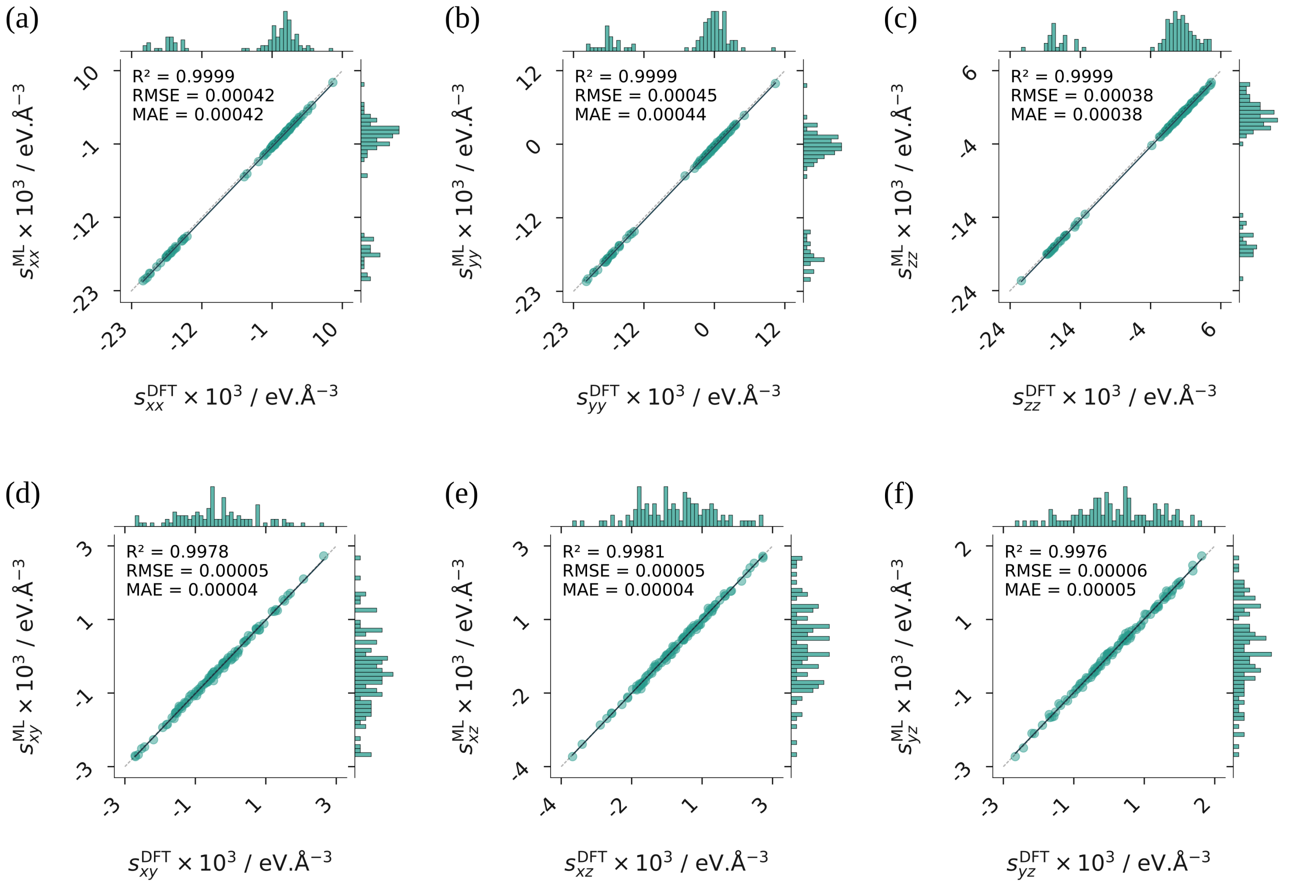

FIG. S5: Correlation plots of the ML-predicted (DFT-computed) stress tensor components (a) s_{xx}^{ML} (s_{xx}^{DFT}), (b) s_{yy}^{ML} (s_{yy}^{DFT}), (c) s_{zz}^{ML} (s_{zz}^{DFT}), (d) s_{xy}^{ML} (s_{xy}^{DFT}), (e) s_{xz}^{ML} (s_{xz}^{DFT}), and (f) s_{yz}^{ML} (s_{yz}^{DFT}), from the testing dataset of Xe@CC3@TBA. The dashed line along the diagonal corresponds to $x = y$, while the solid line represents the linear least-squares regression fit. For each plot, the R-squared, RMSE, and MAE values are given in the upper left corner. Histograms along the axes show the projected distributions of ML-predicted and DFT-computed values from the testing dataset.

II. TRANSFERABILITY OF THE MLIP

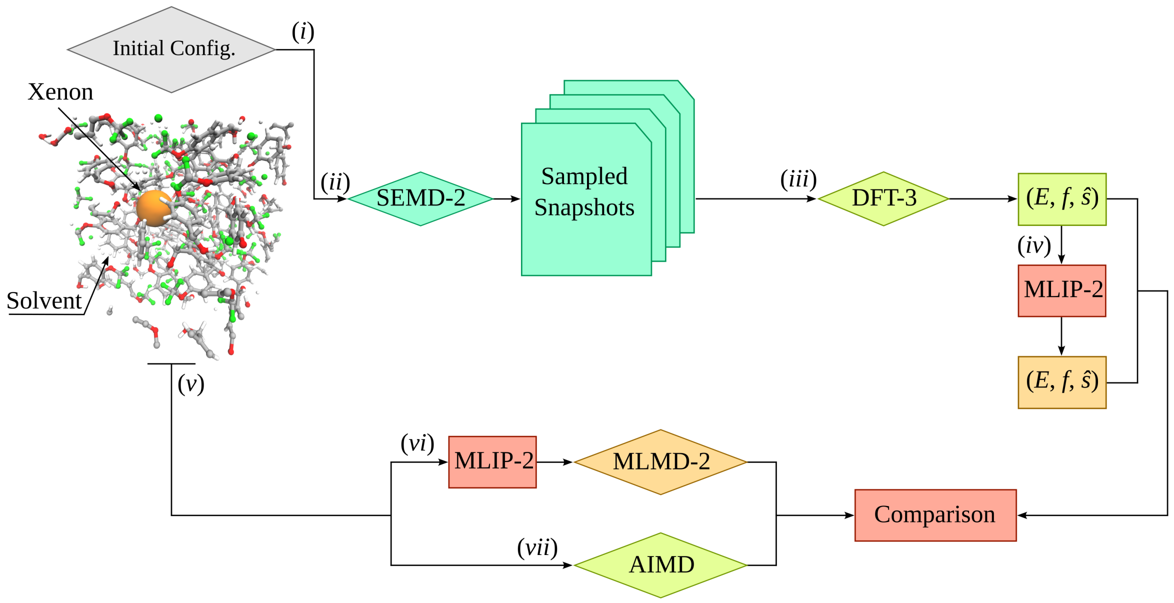

FIG. S6: Diagrammatic representation of the transferability tests workflow, including (i) building the initial Xe@TBA configuration, (ii) performing the second round of SEMD simulations (SEMD-2) and sampling snapshots, (iii) performing single-point PBE-D4 calculations on the sampled snapshots to compute E, $\vec{f}$, and $\boldsymbol{s}$, which, together with the snapshots, constitute the transferability dataset (DFT-3), (iv) computing E, $\vec{f}$, and $\boldsymbol{s}$ using MLIP-2, (v)-(vi) using the initial Xe@TBA configuration and MLIP-2 to perform the second round of MLMD (MLMD-2), and (vii) running *ab initio* MD (AIMD) simulation.

II.1. The Initial Xe@TBA Configuration

The initial Xe@TBA configuration was build using the same method used for Xe@CC3@TBA (**Section I.1**), yielding a density of 1.33 g.mL^{-1} with a 1:50 Xe-to-TBA ratio.

II.2. Semi-Empirical Molecular Dynamics

The second round of SEMD simulations (SEMD-2) were performed on Xe@TBA, and 247 snapshots were sampled (197 at 300 K and 50 at 600 K). These SEMD-2 simulations were carried out under the same conditions as SEMD-1 (**Section I.2**). The system, comprising 1001 atoms in total, was modeled in a 23×23×23 Å^3 periodic box.

II.3. Single-point calculations and *Ab Initio* Molecular Dynamics

Single-point and AIMD calculations were carried out using VASP (version 6.4.1). The former is performed on the sampled SEMD-2 snapshots and the latter is conducted on the Xe@TBA initial configuration. To ensure consistency, both single-point and AIMD calculations employed

the same electronic structure methodology used in **Section I.3**, including identical PAW potentials, basis set, energy cutoff, k-point mesh, and convergence criterion. The AIMD simulation was carried out at 300 K using a NVT ensemble with Nosé–Hoover thermostat and a timestep of 1 fs. Due to high computational cost, the simulations were limited to a total duration of 1 ps. Although full equilibration could not be achieved within this short timescale, the thermostat temperature stabilized after approximately 200 fs.

II.4. Machine Learning Molecular Dynamics

The second round of MLMD simulations (MLMD-2) was performed on Xe@TBA (1001 atoms in a 23×23×23 Å^3 periodic box), and used the same conditions as MLMD-1 (**Section I.5**) except for the simulation length which was 10 ps.

II.5. PCA and t-SNE

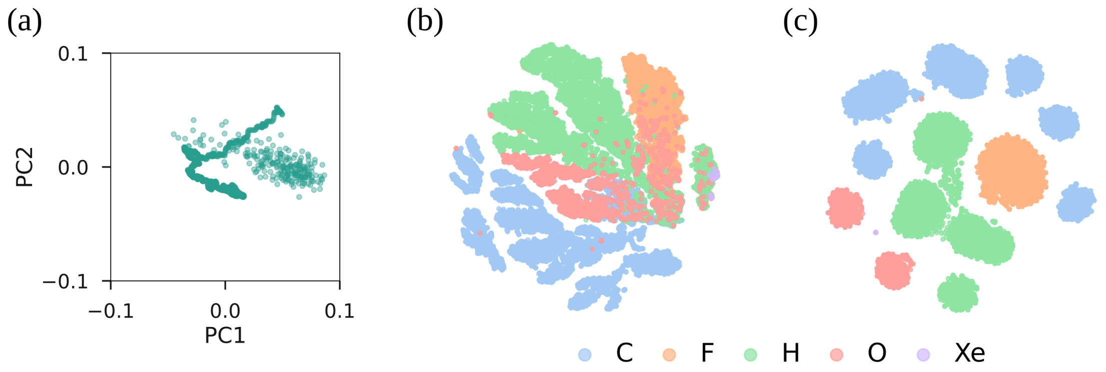

FIG. S7: PCA of DFT-3 dataset in the first two principal components (PC1, PC2) based on structure-averaged SOAP descriptors (a). t-SNE of 3×10^4 randomly sampled individual atomic neighborhoods from the DFT-3 datasets using Cartesian (b) and SOAP (c) representations, with points colored by atomic species.

The two first principle components capture 64.7% of the total variance (PC1: 49.1%, PC2: 15.6%), demonstrating a moderate-range conformational space across both components with PC1 capturing the dominant mode of structural variations (**Figure S7a**).

The same t-SNE features observed previously for DFT-2 dataset, are observed here (**Figures S7b** and **S7c**).

II.6. MLIP predictions: Xe@TBA at RT and 600 K

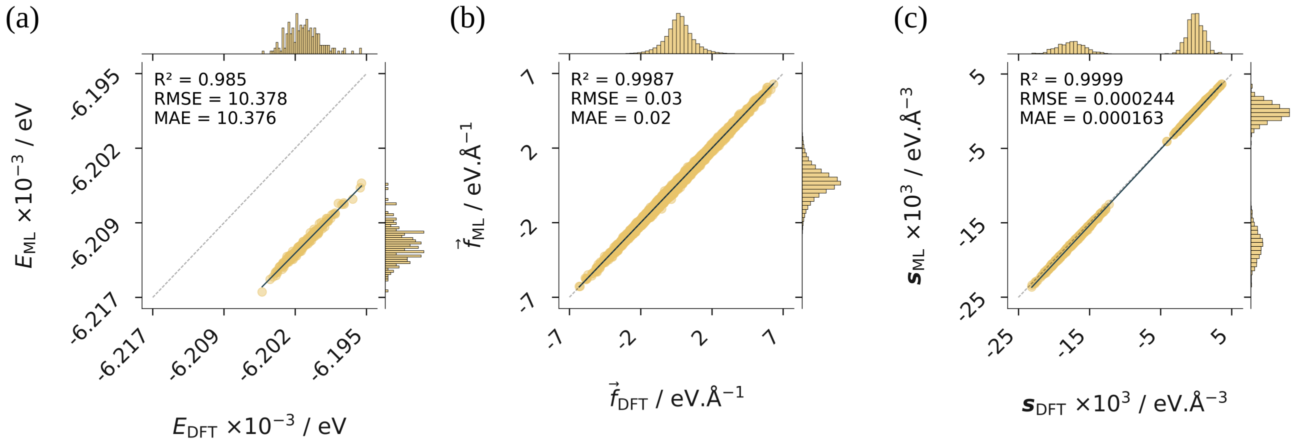

FIG. S8: Correlation plots of the ML-predicted (DFT-computed) (a) E_{ML} (E_{DFT}), (b) $\vec{f}_{\mathrm{ML}}$ ($\vec{f}_{\mathrm{DFT}}$), and (c) $\boldsymbol{s}_{\mathrm{ML}}$ ($\boldsymbol{s}_{\mathrm{DFT}}$), from the testing dataset of Xe@TBA at RT. The dashed line along the diagonal corresponds to $x = y$, while the solid line represents the linear least-squares regression fit. For each plot, the R-squared, RMSE, and MAE values are given in the upper left corner. Histograms along the axes show the projected distributions of ML-predicted and DFT-computed values from the testing dataset.

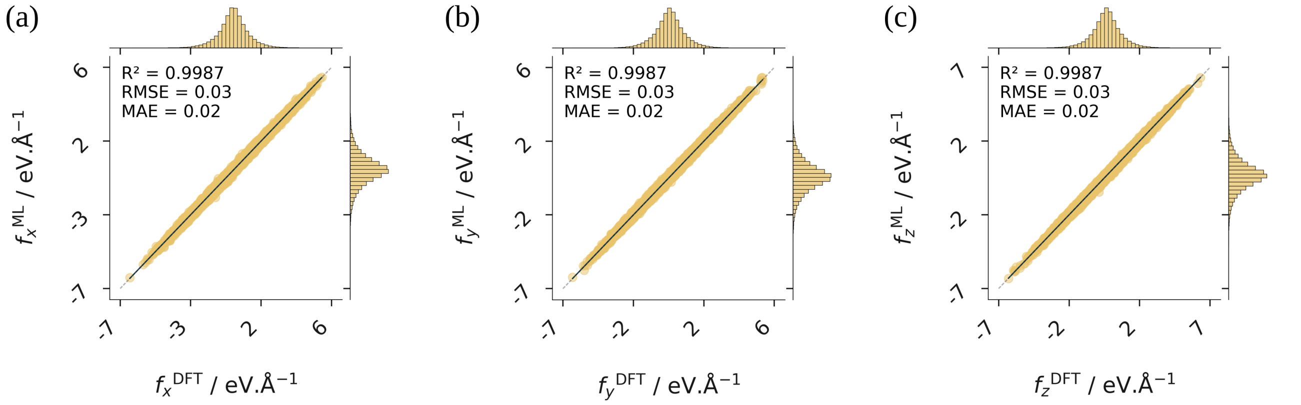

FIG. S9: Correlation plots of the ML-predicted (DFT-computed) atomic force components (a) f_x^{ML} (f_x^{DFT}), (b) f_y^{ML} (f_y^{DFT}), and (c) f_z^{ML} (f_z^{DFT}), from the testing dataset of Xe@TBA at RT. The dashed line along the diagonal corresponds to $x = y$, while the solid line represents the linear least-squares regression fit. For each plot, the R-squared, RMSE, and MAE values are given in the upper left corner. Histograms along the axes show the projected distributions of ML-predicted and DFT-computed values from the testing dataset.

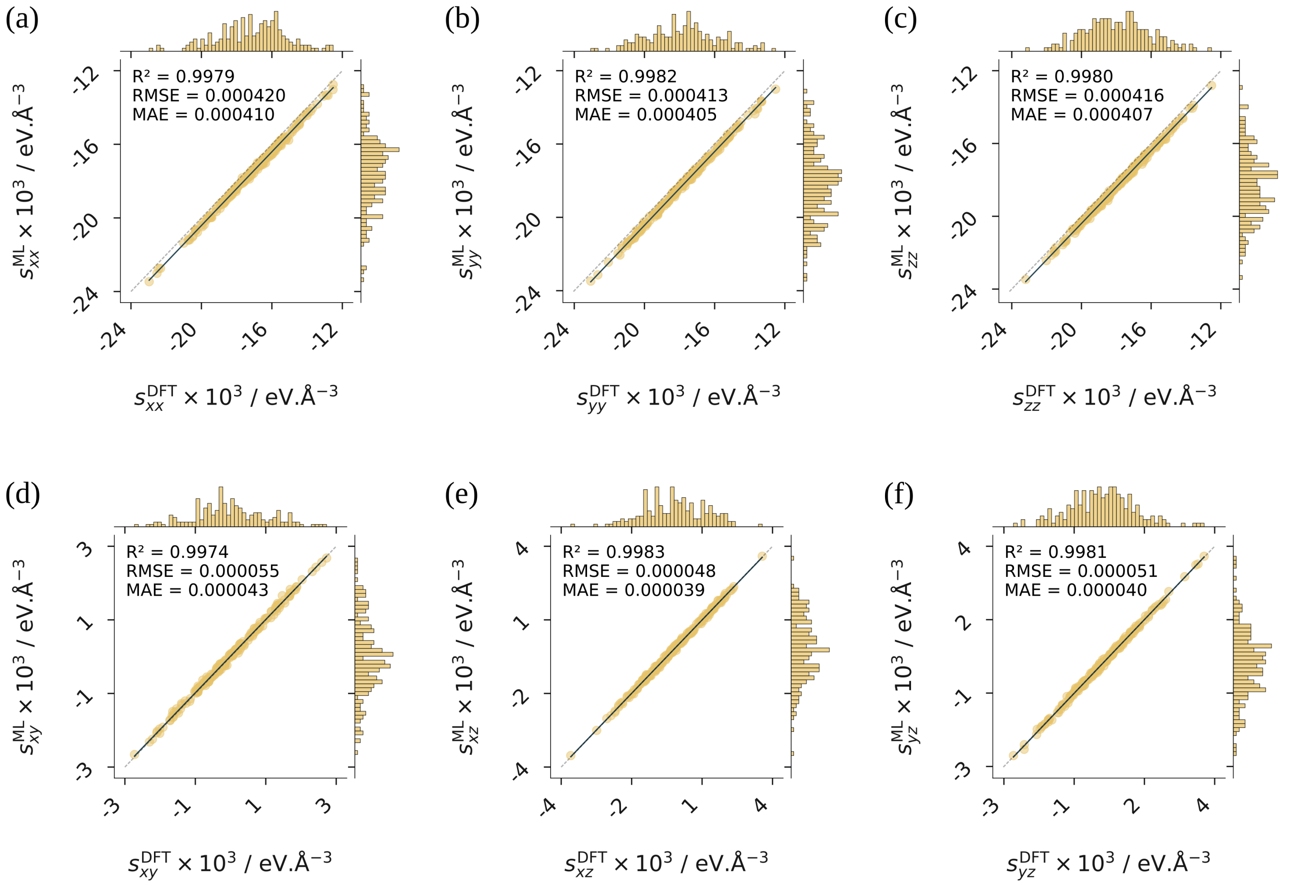

FIG. S10: Correlation plots of the ML-predicted (DFT-computed) stress tensor components (a) s_{xx}^{ML} (s_{xx}^{DFT}), (b) s_{yy}^{ML} (s_{yy}^{DFT}), (c) s_{zz}^{ML} (s_{zz}^{DFT}), (d) s_{xy}^{ML} (s_{xy}^{DFT}), (e) s_{xz}^{ML} (s_{xz}^{DFT}), and (f) s_{yz}^{ML} (s_{yz}^{DFT}), from the testing dataset of Xe@TBA at RT. The dashed line along the diagonal corresponds to $x = y$, while the solid line represents the linear least-squares regression fit. For each plot, the R-squared, RMSE, and MAE values are given in the upper left corner. Histograms along the axes show the projected distributions of ML-predicted and DFT-computed values from the testing dataset.

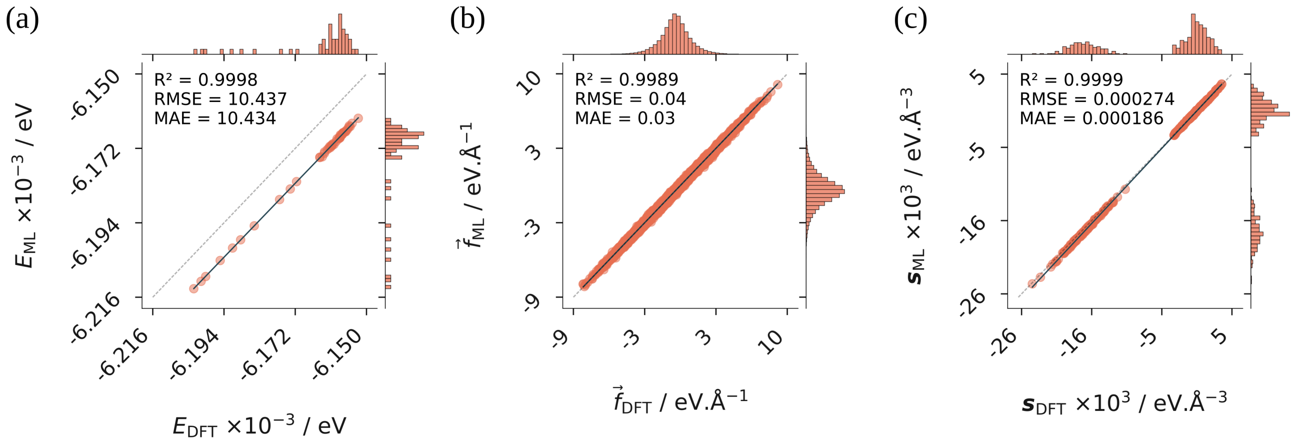

FIG. S11: Correlation plots of the ML-predicted (DFT-computed) (a) E_{ML} (E_{DFT}), (b) $\vec{f}_{\mathrm{ML}}$ ($\vec{f}_{\mathrm{DFT}}$), and (c) $\boldsymbol{s}_{\mathrm{ML}}$ ($\boldsymbol{s}_{\mathrm{DFT}}$), from the testing dataset of Xe@TBA at 600 K. The dashed line along the diagonal corresponds to $x = y$, while the solid line represents the linear least-squares regression fit. For each plot, the R-squared, RMSE, and MAE values are given in the upper left corner. Histograms along the axes show the projected distributions of ML-predicted and DFT-computed values from the testing dataset.

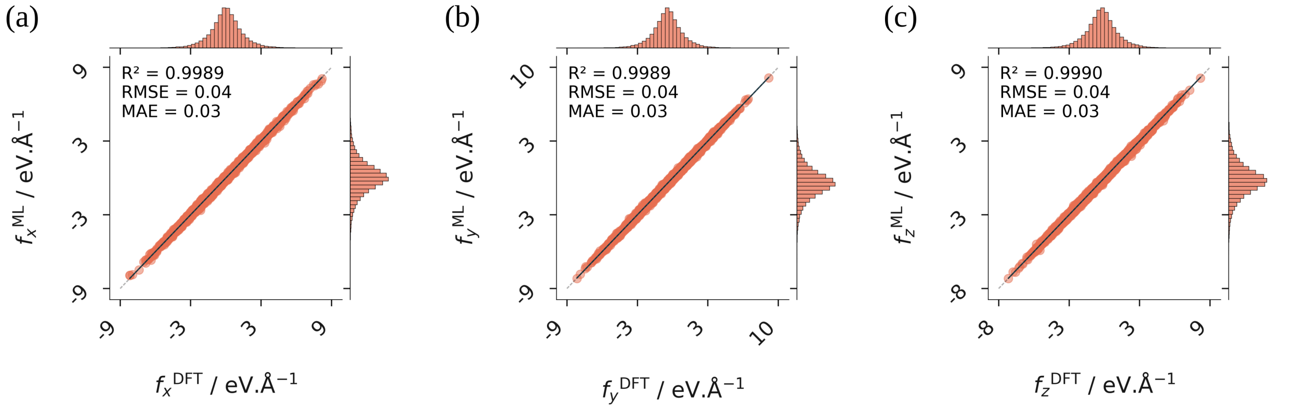

FIG. S12: Correlation plots of the ML-predicted (DFT-computed) atomic force components (a) f_x^{ML} (f_x^{DFT}), (b) f_y^{ML} (f_y^{DFT}), and (c) f_z^{ML} (f_z^{DFT}), from the testing dataset of Xe@TBA at 600 K. The dashed line along the diagonal corresponds to $x = y$, while the solid line represents the linear least-squares regression fit. For each plot, the R-squared, RMSE, and MAE values are given in the upper left corner. Histograms along the axes show the projected distributions of ML-predicted and DFT-computed values from the testing dataset.

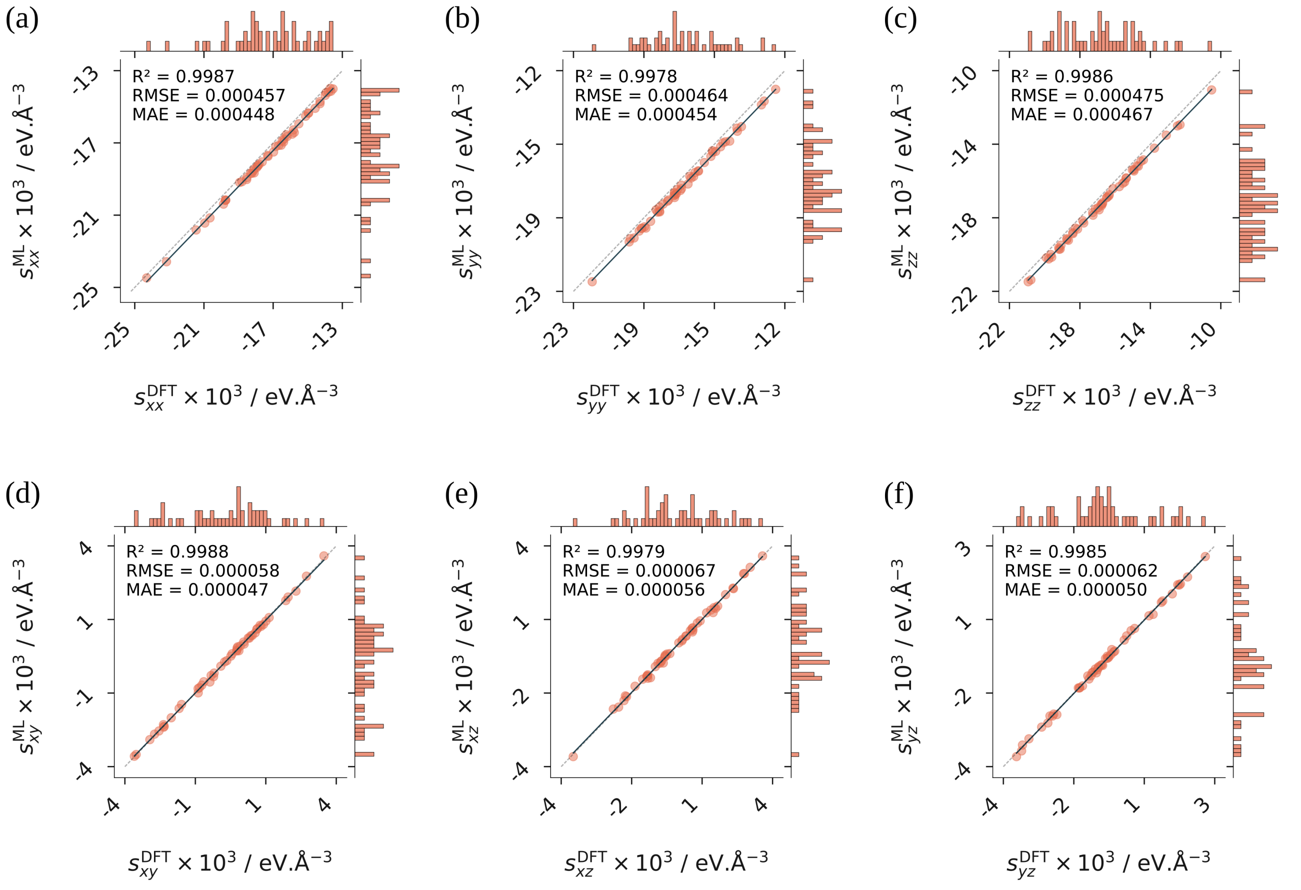

FIG. S13: Correlation plots of the ML-predicted (DFT-computed) stress tensor components (a) s_{xx}^{ML} (s_{xx}^{DFT}), (b) s_{yy}^{ML} (s_{yy}^{DFT}), (c) s_{zz}^{ML} (s_{zz}^{DFT}), (d) s_{xy}^{ML} (s_{xy}^{DFT}), (e) s_{xz}^{ML} (s_{xz}^{DFT}), and (f) s_{yz}^{ML} (s_{yz}^{DFT}), from the testing dataset of Xe@TBA at 600 K. The dashed line along the diagonal corresponds to $x = y$, while the solid line represents the linear least-squares regression fit. For each plot, the R-squared, RMSE, and MAE values are given in the upper left corner. Histograms along the axes show the projected distributions of ML-predicted and DFT-computed values from the testing dataset.

II.7. Radial Distribution Function

The radial distribution function $g(r)$ was computed every 1 fs for AIMD and every 10 fs for MLMD-2 over trajectories of 1 ps and 10 ps, respectively (corresponding to 1000 snapshots in each case). Calculations were performed for all possible atom pairs as well as for specific Xe–H, Xe–C, Xe–O, and Xe–F pairs using OVITO software (version 3.11.3).[37] For each snapshot, total and pair-specific $g(r)$ were computed over 0–10 Å, using 1000 bins for the total and 50 bins for the pair-specific distributions. The resulting $g(r)$ data were smoothed with a Savitzky–Golay filter,[38] applying a window length of 5 points for the total and 10 points for the pair-specific $g(r)$, with a polynomial order of 1.

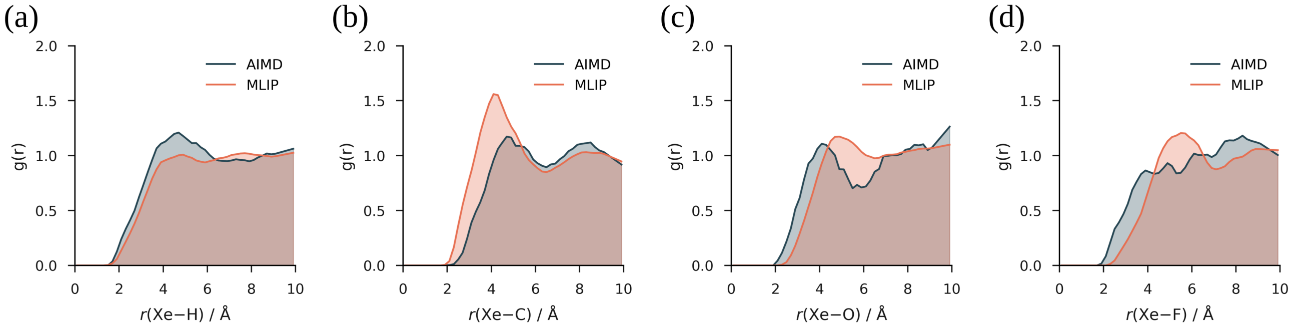

FIG. S14: Pair-specific $g(r)$ as a function of the interatomic distance, r, for Xe−H (a), Xe−C (b), Xe−O (c), and Xe−F (d) pairs, from AIMD (dark blue) and MLMD-2 (orange) simulation trajectories.

III. THE NUCLEAR MAGNETIC RESONANCE MACHINE LEARNING MODEL

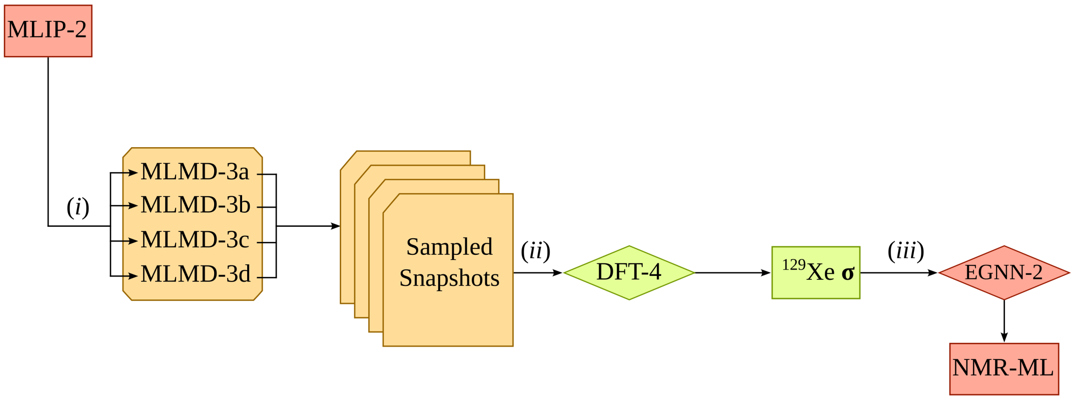

FIG. S15: Diagrammatic representation of the NMR machine learning (NMR-ML) model, which include (*i*) running the third round of MLMD simulations (MLMD-3a to 3d) using MLIP-2 and sample snapshots, (*ii*) computing the ^{129}Xe magnetic shielding tensor, $\boldsymbol{\sigma}$, for these snapshots (DFT-4), and (*iii*) training a second EGNN model (EGNN-2) on the ^{129}Xe $\boldsymbol{\sigma}$ values to obtain the NMR-ML model.

III.1. Machine Learning Molecular Dynamics

The third round of MLMD simulations (MLMD-3) was performed on Xe@CC3@TBA (1170 atoms in a 23.9×23.9×23.9 Å^3 periodic box) using the same conditions used in **Section I.5**, they included four simulations carried out at 260 K (MLMD-3a), 280 K (MLMD-3b), 300 K (MLMD-3c), and 320 K (MLMD-3d). Each simulation began with a 0.1 ns equilibration phase, followed by production runs of 9.9 ns. A total of 3125 snapshots were sampled from these simulations.

III.2. ^{129}Xe $\boldsymbol{\sigma}$ Calculations

The ^{129}Xe magnetic shielding tensor, $\boldsymbol{\sigma}$, was computed using Turbomole (V7-8),[39–42] following the methodology described in ref([3]). It was computed using DFT for the sampled MLMD-3 snapshots, with the exact-two-component (X2C) scalar-relativistic Hamiltonian.[43] Three sets of calculations were carried out, with Xe described in all cases using x2c-TZVPall-s basis set,[44] while the remaining atoms (H, C, N, O, and F) were treated with different basis sets. These calculations use (*i*) PBE functional with x2c-SVPall basis set (PBE/SVP),[45] (*ii*) PBE functional with x2c-TZVPall basis set (PBE/TZVP), and (*iii*) BHandHLYP functional[46–48] with x2c-SVPall basis set (BHandHLYP/SVP). The resolution-of-identity approximation with the corresponding auxiliary basis sets was employed. Long-range dispersion interactions were treated with the semi-empirical D4 correction.[11,12] Since only $\boldsymbol{\sigma}$ of Xe was of interest, it was not computed for the other atoms.

The final ^{129}Xe $\boldsymbol{\sigma}$ was obtained using the relation:

$$\boldsymbol{\sigma} = \boldsymbol{\sigma}_{\mathrm{BHandHLYP/SVP}} + \boldsymbol{\sigma}_{\mathrm{PBE/TZVP}} - \boldsymbol{\sigma}_{\mathrm{PBE/SVP}}. \quad (1)$$

The isotropic magnetic shielding, σ_{iso}, was calculated as:

$$\sigma_{\text{iso}} = \frac{1}{3}\text{Tr}(\boldsymbol{\sigma}) = \frac{1}{3}(\sigma_{xx} + \sigma_{yy} + \sigma_{zz}), \tag{2}$$

and the isotropic chemical shift, δ_{iso}, is given as:

$$\delta_{\text{iso}} = \sigma_{\text{iso}}^{\text{ref}} - \sigma_{\text{iso}}, \tag{3}$$

where $\sigma_{\text{iso}}^{\text{ref}}$ is the reference isotropic magnetic shielding value, computed as 5847.6 ppm using DFT by considering a single Xe atom in vacuum. The DFT calculation used X2C scalar-relativistic Hamiltonian with BHandHLYP functional and x2c-TZVPall-s basis set.

III.3. PCA and t-SNE

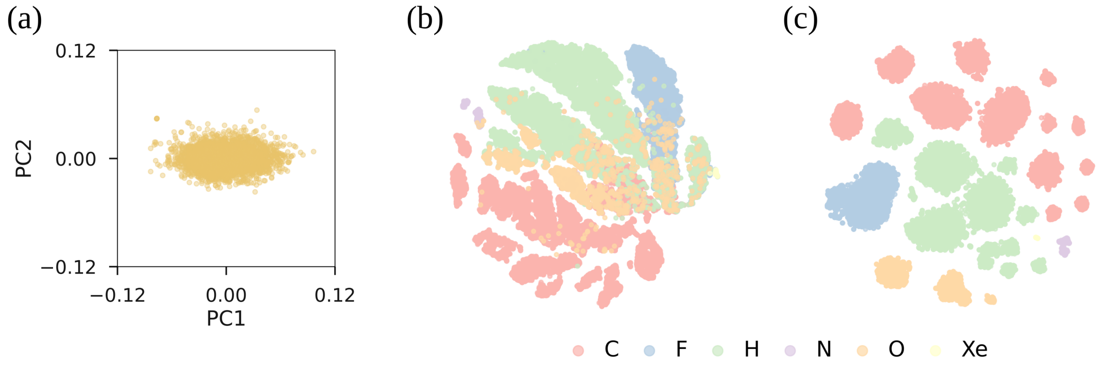

FIG. S16: PCA of DFT-4 dataset in the first two principal components (PC1, PC2) based on structure-averaged SOAP descriptors (a). t-SNE of 3×10^4 randomly sampled individual atomic neighborhoods from the DFT-4 datasets using Cartesian (b) and SOAP (c) representations, with points colored by atomic species.

The two first principle components capture 55.7% of the total structural variance (PC1: 46.6%, PC2: 9.1%), which, while lower than that of the previous datasets, provides sufficient coverage of Xe local environments (**Figure S16a**).

The same t-SNE features observed previously for DFT-2 and DFT-3 datasets, are observed here (**Figures S16b** and **S16c**).

III.4. The NMR-ML Neural Network Architecture

We train *MatTen*,[49,50] a recently developed $E(3)$-EGGN[16] designed for predicting atomic and structure tensors, to obtain the NMR-ML model for predicting ^{129}Xe σ in Xe@CC3@TBA. *MatTen* uses equivariant message passing to directly predict rank-2 tensors while preserving rotational and translational symmetries, making it particularly well-suited for NMR chemical shift tensor prediction.

The *MatTen* model (version 0.0.1) operates with an r_{max} of 5 Å, employing Bessel radial basis functions with 8 basis functions spanning from 0 to 5 Å. Spherical harmonics embeddings for edge directions are represented as $0e + 1o + 2e$ irreducible representations under full $O(3)$ symmetry. The atomic species are embedded in a 16-dimensional space, with each atom type

mapped accordingly. The network consists of three message passing convolutional layers with convolution layer irreducible representations of $320o + 320e + 161o + 161e + 42o + 42e$. The radial network employs two invariant layers with 32 hidden neurons each. Gate-type nonlinearity with batch normalization is used throughout the network, with residual connections (ResNet) enabled to improve training stability and convergence.

Similar to the MLIP (**Section I.4**), training was performed following a 8:1:1 dataset split, *i.e.*, 2500, 312, and 312 snapshots for training validation, and testing, respectively. The ^{129}Xe $\boldsymbol{\sigma}$ tensor was symmetrized before the training, *i.e.* the target tensor follows the symmetric tensor formula $ij = ji$. Each structure contains atomic coordinates with an atom selector specifically targeting ^{129}Xe atoms for tensor prediction. The average number of neighbors was automatically determined from the training set for proper normalization.

Optimization was conducted using the *Pytorch* (version 2.0.1) *Adam* optimizer at an initial learning rate of 0.01 and weight decay of 10^{-5}. A "*ReduceLROnPlateau*" learning rate scheduler was applied with a patience of 50 epochs and a decay factor of 0.5. Training was continued for a maximum of 5000 epochs with early stopping criteria based on validation score with a patience of 150 epochs. Batch size was set to 1 with data shuffling enabled during training.

The model outputs tensors in irreducible representation (irreps) format with the constraint $ij = ji$. Training was performed on a single NVIDIA V100 GPU with a total memory of 32 GB.

III.5. Training and Validation Progress, and Tests of the NMR-ML Model

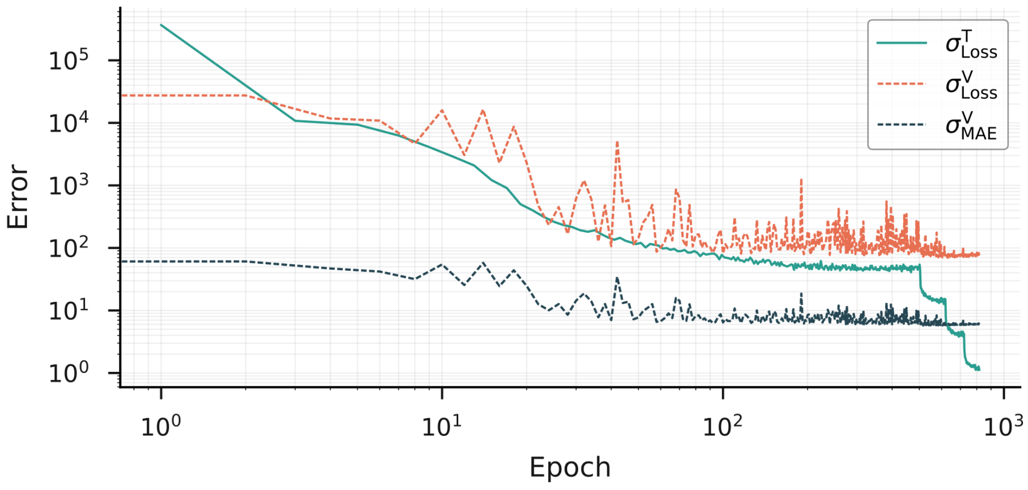

FIG. S17: Training and validation progress from *MatTen* for Xe@CC3@TBA. The figure illustrates the evolution of the validation MAE for ^{129}Xe σ (σ^{V}_{MAE}, ppm), and the loss function for the training (σ^{T}_{Loss}, ppm) and validation (σ^{V}_{Loss}, ppm).

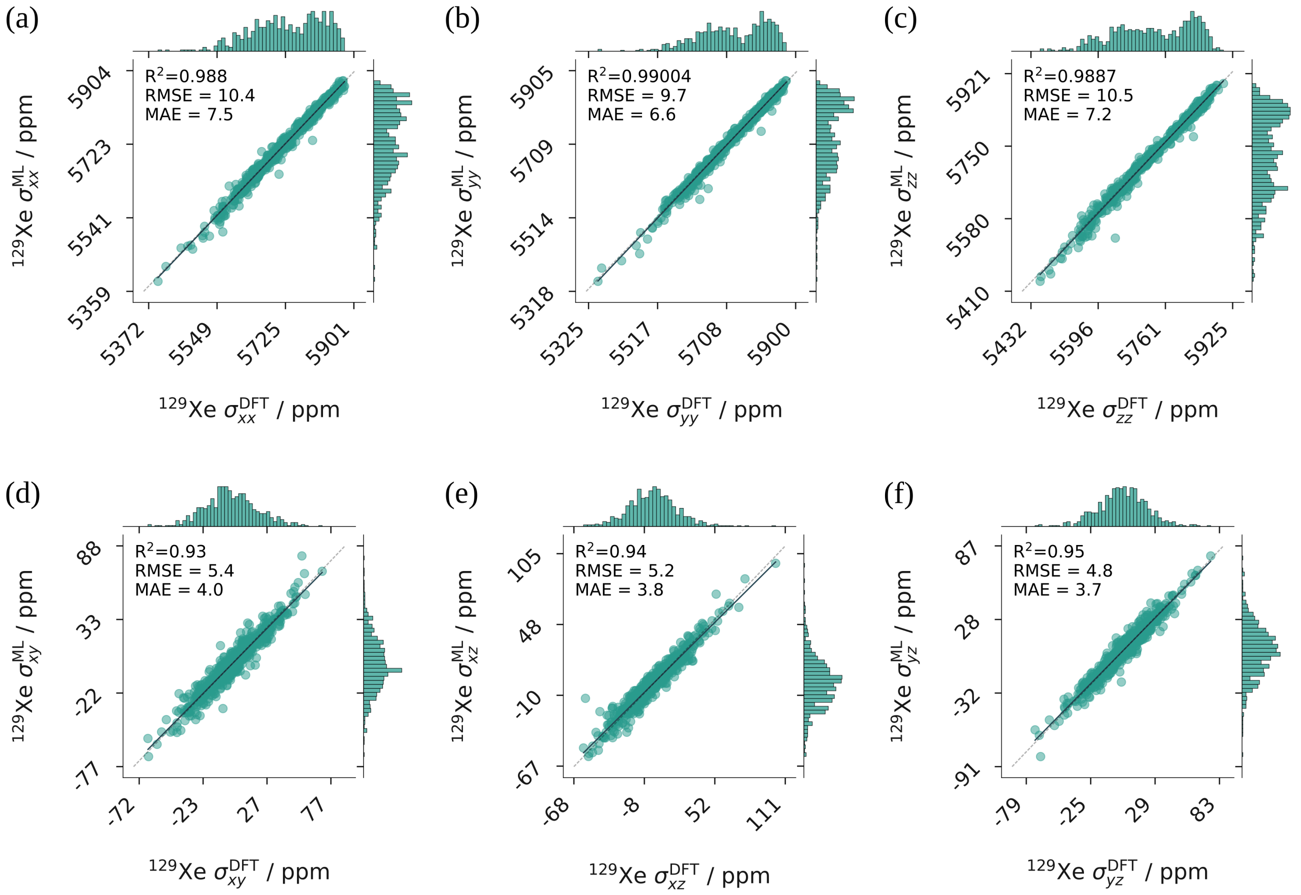

FIG. S18: Correlation plots of the ML-predicted (DFT-computed) ^{129}Xe magnetic shielding tensor components (a) $\sigma_{xx}^{\rm ML}$ ($\sigma_{xx}^{\rm DFT}$), (b) $\sigma_{yy}^{\rm ML}$ ($\sigma_{yy}^{\rm DFT}$), (c) $\sigma_{zz}^{\rm ML}$ ($\sigma_{zz}^{\rm DFT}$), (d) $\sigma_{xy}^{\rm ML}$ ($\sigma_{xy}^{\rm DFT}$), (e) $\sigma_{xz}^{\rm ML}$ ($\sigma_{xz}^{\rm DFT}$), and (f) $\sigma_{yz}^{\rm ML}$ ($\sigma_{yz}^{\rm DFT}$), from the testing dataset of Xe@CC3@TBA. The dashed line along the diagonal corresponds to $x = y$, while the solid line represents the linear least-squares regression fit. For each plot, the R-squared, RMSE, and MAE values are given in the upper left corner. Histograms along the axes show the projected distributions of ML-predicted and DFT-computed values from the testing dataset.

IV. PRODUCTION SIMULATIONS

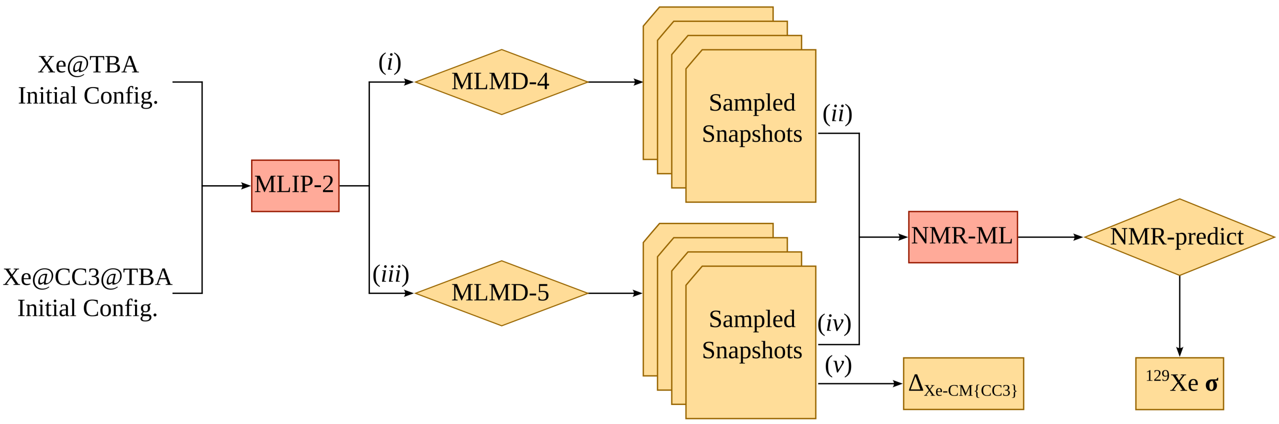

FIG. S19: Diagrammatic representation of the production simulations, which include (*i*) using MLIP-2 to run the fourth round, production run, MLMD simulations (MLMD-4) and sample snapshots for Xe@TBA, (*ii*) compute ^{129}Xe $\boldsymbol{\sigma}$ with the NMR-ML model for the sampled MLMD-4 snapshots, (*iii*) using MLIP-2 to run the fifth round, production run, MLMD simulations (MLMD-5) and sample snapshots for Xe@CC3@TBA, (*iv*) compute ^{129}Xe $\boldsymbol{\sigma}$ with the NMR-ML model for the sampled MLMD-5 snapshots, and (*v*) compute $\Delta_{\text{Xe-CM\{CC3\}}}$ for the sampled MLMD-5 snapshots.

IV.1. Machine Learning Molecular Dynamics

The fourth and fifth rounds of MLMD simulations (MLMD-4 and MLMD-5) were performed on Xe@TBA (1001 atoms in a 23×23×23 Å^3 periodic box) and a 2×2×2 supercell of Xe@CC3@TBA (9360 atoms in a 47.7×47.7×47.7 Å^3 periodic box), respectively. These simulations used the same conditions in **Section I.5**, they were performed at 300 K with a 0.1 ns equilibration phase, followed by production runs of 5 and 1 ns for Xe@TBA and Xe@CC3@TBA, respectively.

IV.2. Properties Simulations and Data Analysis

The distance between xenon and the CC3 cage center of mass (CM) at time t, $\Delta_{\text{Xe-CM\{CC3\}}}$ (Å), is computed considering two steps. We first need to calculate the CM of the CC3 cage at a time t, which is given as the weighted average of all constituent atoms and defined by:

$$\vec{r}_{\text{COM}}^{\,\text{CC3}} = \frac{\sum_{i=1}^{N_{\text{CC3}}} m_i \vec{r}_i}{\sum_{i=1}^{N_{\text{CC3}}} m_i}, \tag{4}$$

Where N_{CC3} is the number of atoms forming the CC3 cage (168 atoms), m_i is the atomic mass of atom i, and $\vec{r}_i$ is the position vector of atom i at time t.

Now we compute $\Delta_{\text{Xe-CM\{CC3\}}}$ using the minimum image convention to properly account for periodic boundary conditions using:

$$\Delta_{\text{Xe-CM\{CC3\}}} = \text{MIC}\left(\vec{r}_{\text{Xe}} - \vec{r}^{\,\text{CC3}}_{\text{COM}}, \mathbf{L}\right) , \tag{5}$$

Where $\vec{r}_{\text{Xe}}$ is the xenon atom position at a time t, and $\text{MIC}(\vec{d}, \mathbf{L})$ denotes the minimum image convention function that accounts for the simulation cell matrix $\mathbf{L}$.

For MLMD-4 and MLMD-5, $\Delta_{\text{Xe-CM\{CC3\}}}$ and ^{129}Xe δ_{iso} were computed every 1 and 0.1 ps, respectively.

$\Delta_{\text{Xe-CM\{CC3\}}}$ and ^{129}Xe δ_{iso} were obtained using ASE code (version 3.22.1) combined with NumPy library (version 1.26.4)[51] and a Python-based *MatTen* predictor (from this work), respectively. The resulting data were smoothed with a Savitzky–Golay filter, with a window length of 81 points and polynomial order of 3. All analyses and visualizations were carried out in Python using NumPy (version 1.26.4), Matplotlib (version 3.8.4),[52] Seaborn (version 0.13.2),[53] and VMD (version 1.9.3).[54,55]

IV.3. The Production Machine Learning Molecular Dynamics Simulation

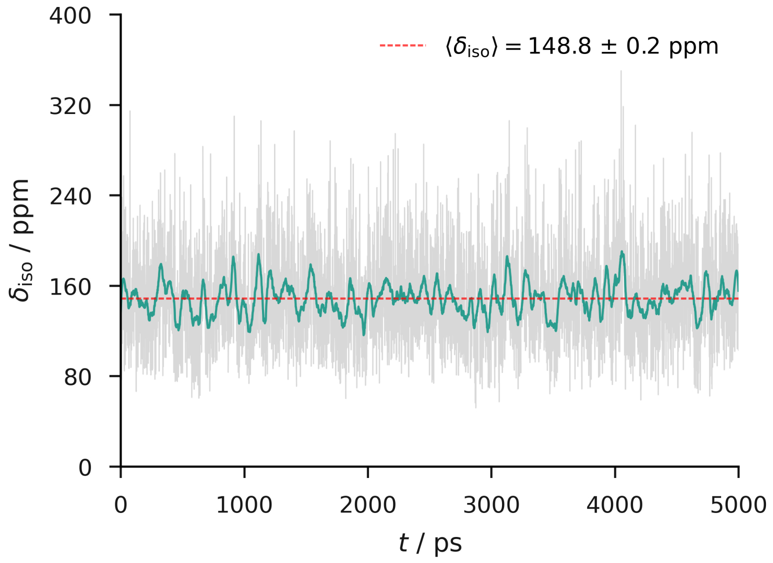

FIG. S20: Evolution with respect to time of ^{129}Xe δ_{iso} for Xe@TBA from the MLMD-4 trajectory. The raw and filtered data is plotted in gray and teal, respectively. The average ^{129}Xe δ_{iso} ($\langle\delta_{\text{iso}}\rangle$, ppm) is given and highlighted in red.

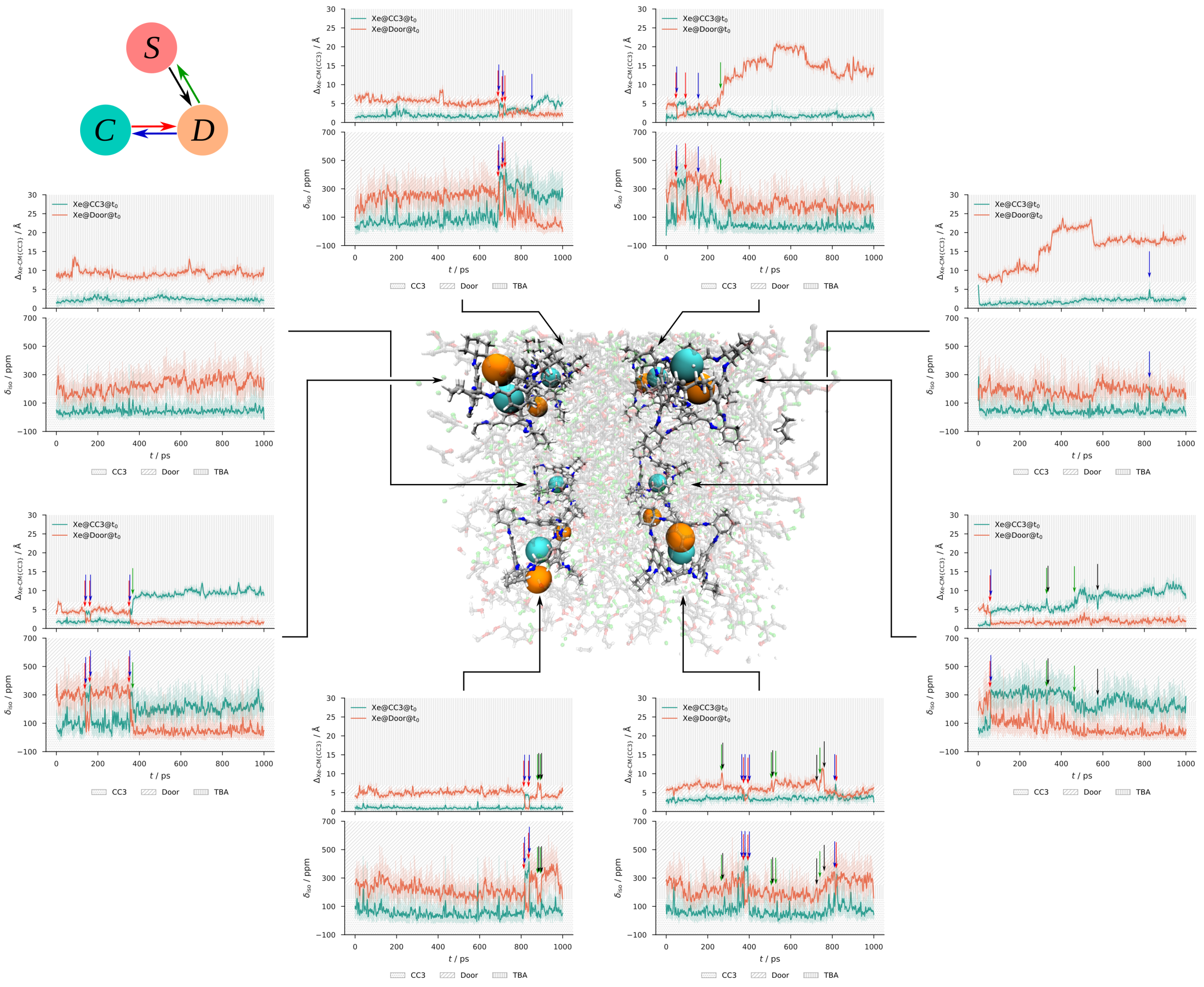

FIG. S21: Evolution of the displacement of Xe from the center of mass (CM) of the CC3 cage ($\Delta_{\text{Xe-CM\{CC3\}}}$, Å) and ^{129}Xe isotropic chemical shift (δ_{iso}, ppm) as a function of time (t, ps) for each of the eight CC3 cages in Xe@CC3@TBA. In each plot, the y-axis is divided into three patterned regions where Xe is at C (dotted), D (///), and S (|||). Raw and filtered data are shown as semi-transparent and opaque lines, respectively. Xenon atoms initially at C and D are labeled Xe@CC3@t$_0$ (teal) and Xe@Door@t$_0$ (orange), respectively, with t_0 referring to their initial locations. Exchange events, where the two Xe atoms exchange sites, are indicated by colored arrows (top-left corner). Black arrows link each plot to its corresponding CC3 cages in the perspective view of Xe@CC3@TBA structure (center).

REFERENCES

(1) Martínez, L.; Andrade, R.; Birgin, E. G.; Martínez, J. M. PACKMOL: A Package for Building Initial Configurations for Molecular Dynamics Simulations. *J. Comput. Chem.* **2009**, *30*, 2157–2164, DOI: 10.1002/jcc.21224.

(2) Baerends, E. J.; Aguirre, N. F.; Austin, N. D.; Autschbach, J.; Bickelhaupt, F. M.; Bulo, R.; Cappelli, C.; van Duin, A. C. T.; Egidi, F.; Fonseca Guerra, C.; Förster, A.; Franchini, M.; Goumans, T. P. M.; Heine, T.; Hellström, M.; Jacob, C. R.; Jensen, L.; Krykunov, M.; van Lenthe, E.; Michalak, A.; Mitoraj, M. M.; Neugebauer, J.; Nicu, V. P.; Philipsen, P.; Ramanantoanina, H.; Rüger, R.; Schreckenbach, G.; Stener, M.; Swart, M.; Thijssen, J. M.; Trnka, T.; Visscher, L.; Yakovlev, A.; van Gisbergen, S. The Amsterdam Modeling Suite. *J. Chem. Phys.* **2025**, *162*, 162501, DOI: 10.1063/5.0258496.

(3) Mailhiot, S. E.; Peuravaara, P.; Egleston, B. D.; Kearsey, R. J.; Mareš, J.; Komulainen, S.; Selent, A.; Kantola, A. M.; Cooper, A. I.; Vaara, J.; Greenaway, R. L.; Lantto, P.; Telkki, V.-V. Gas Uptake and Thermodynamics in Porous Liquids Elucidated by ^{129}Xe NMR. *J. Phys. Chem. Lett.* **2024**, *15*, 5323–5330, DOI: 10.1021/acs.jpclett.4c00223.

(4) Hourahine, B.; Aradi, B.; Blum, V.; Bonafé, F.; Buccheri, A.; Camacho, C.; Cevallos, C.; Deshaye, M. Y.; Dumitrică, T.; Dominguez, A.; Ehlert, S.; Elstner, M.; van der Heide, T.; Hermann, J.; Irle, S.; Kranz, J. J.; Köhler, C.; Kowalczyk, T.; Kubař, T.; Lee, I. S.; Lutsker, V.; Maurer, R. J.; Min, S. K.; Mitchell, I.; Negre, C.; Niehaus, T. A.; Niklasson, A. M. N.; Page, A. J.; Pecchia, A.; Penazzi, G.; Persson, M. P.; Řezáč, J.; Sánchez, C. G.; Sternberg, M.; Stöhr, M.; Stuckenberg, F.; Tkatchenko, A.; Yu, V. W.-z.; Frauenheim, T. DFTB+, a Software Package for Efficient Approximate Density Functional Theory Based Atomistic Simulations. *J. Chem. Phys.* **2020**, *152*, 124101, DOI: 10.1063/1.5143190.

(5) Grimme, S.; Bannwarth, C.; Shushkov, P. A Robust and Accurate Tight-Binding Quantum Chemical Method for Structures, Vibrational Frequencies, and Noncovalent Interactions of Large Molecular Systems Parametrized for All spd-Block Elements (Z = 1–86). *J. Chem. Theory Comput.* **2017**, *13*, 1989–2009, DOI: 10.1021/acs.jctc.7b00118.

(6) Bannwarth, C.; Ehlert, S.; Grimme, S. GFN2-xTB—An Accurate and Broadly Parametrized Self-Consistent Tight-Binding Quantum Chemical Method with Multipole Electrostatics and Density-Dependent Dispersion Contributions. *J. Chem. Theory Comput.* **2019**, *15*, 1652–1671, DOI: 10.1021/acs.jctc.8b01176.

(7) Martyna, G. J.; Klein, M. L.; Tuckerman, M. Nosé–Hoover Chains: The Canonical Ensemble via Continuous Dynamics. *J. Chem. Phys.* **1992**, *97*, 2635–2643, DOI: 10.1063/1.463940.

(8) Kresse, G.; Furthmüller, J. Efficiency of Ab-Initio Total Energy Calculations for Metals and Semiconductors Using a Plane-Wave Basis Set. *Comput. Mater. Sci.* **1996**, *6*, 15–50, DOI: 10.1016/0927-0256(96)00008-0.

(9) Kresse, G.; Furthmüller, J. Efficient Iterative Schemes for *Ab Initio* Total-Energy Calculations Using a Plane-Wave Basis Set. *Phys. Rev. B* **1996**, *54*, 11169–11186, DOI: 10.1103/PhysRevB.54.11169.

(10) Perdew, J. P.; Burke, K.; Ernzerhof, M. Generalized Gradient Approximation Made Simple. *Phys. Rev. Lett.* **1996**, *77*, 3865–3868, DOI: 10.1103/PhysRevLett.77.3865.

(11) Caldeweyher, E.; Ehlert, S.; Hansen, A.; Neugebauer, H.; Spicher, S.; Bannwarth, C.; Grimme, S. A Generally Applicable Atomic-Charge Dependent London Dispersion Correction. *J. Chem. Phys.* **2019**, *150*, 154122, DOI: 10.1063/1.5090222.

(12) Caldeweyher, E.; Mewes, J.-M.; Ehlert, S.; Grimme, S. Extension and Evaluation of the D4 London-Dispersion Model for Periodic Systems. *Phys. Chem. Chem. Phys.* **2020**, *22*, 8499–8512, DOI: 10.1039/D0CP00502A.

(13) Blöchl, P. E. Projector Augmented-Wave Method. *Phys. Rev. B* **1994**, *50*, 17953–17979, DOI: 10.1103/PhysRevB.50.17953.

(14) Kresse, G.; Joubert, D. From Ultrasoft Pseudopotentials to the Projector Augmented-Wave Method. *Phys. Rev. B* **1999**, *59*, 1758–1775, DOI: 10.1103/PhysRevB.59.1758.

(15) Musaelian, A.; Batzner, S.; Johansson, A.; Sun, L.; Owen, C. J.; Kornbluth, M.; Kozinsky, B. Learning Local Equivariant Representations for Large-Scale Atomistic Dynamics. *Nat. Commun.* **2023**, *14*, 579, DOI: 10.1038/s41467-023-36329-y.

(16) Geiger, M.; Smidt, T. e3nn: Euclidean Neural Networks. *arXiv* **18 Jul 2022**, arXiv:2207.09453 (accessed 2025-10-04), DOI: 10.48550/arXiv.2207.09453.

(17) Bihani, V.; Mannan, S.; Pratiush, U.; Du, T.; Chen, Z.; Miret, S.; Micoulaut, M.; Smedskjaer, M. M.; Ranu, S.; Krishnan, N. M. A. EGraFFBench: Evaluation of Equivariant Graph Neural Network Force Fields for Atomistic Simulations. *Digit. Discov.* **2024**, *3*, 759–768, DOI: 10.1039/D4DD00027G.

(18) Kozinsky, B.; Musaelian, A.; Johansson, A.; Batzner, S. Scaling the Leading Accuracy of Deep Equivariant Models to Biomolecular Simulations of Realistic Size. *Int. Conf. High Perform. Comput. Netw. Storage Anal. SC* **2023**, *2*, 1–12, DOI: 10.1145/3581784.3627041.

(19) Tan, C. W.; Descoteaux, M. L.; Kotak, M.; Nascimento, G. d. M.; Kavanagh, S. R.; Zichi, L.; Wang, M.; Saluja, A.; Hu, Y. R.; Smidt, T.; Johansson, A.; Witt, W. C.; Kozinsky, B.; Musaelian, A. High-Performance Training and Inference for Deep Equivariant Interatomic Potentials. *arXiv* **22 Apr 2025**, arXiv:2504.16068 (accessed 2025-10-04), DOI: 10.48550/arXiv.2504.16068.

(20) Zhu, A.; Batzner, S.; Musaelian, A.; Kozinsky, B. Fast Uncertainty Estimates in Deep Learning Interatomic Potentials. *J. Chem. Phys.* **2023**, *158*, 164111, DOI: 10.1063/5.0136574.

(21) Batzner, S.; Musaelian, A.; Sun, L.; Geiger, M.; Mailoa, J. P.; Kornbluth, M.; Molinari, N.; Smidt, T. E.; Kozinsky, B. E(3)-Equivariant Graph Neural Networks for Data-Efficient and Accurate Interatomic Potentials. *Nat. Commun.* **2022**, *13*, 2453, DOI: 10.1038/s41467-022-29939-5.

(22) Larsen, A. H.; Mortensen, J. J.; Blomqvist, J.; Castelli, I. E.; Christensen, R.; Dułak, M.; Friis, J.; Groves, M. N.; Hammer, B.; Hargus, C.; Hermes, E. D.; Jennings, P. C.; Jensen, P. B.; Kermode, J.; Kitchin, J. R.; Kolsbjerg, E. L.; Kubal, J.; Kaasbjerg, K.; Lysgaard, S.; Maronsson, J. B.; Maxson, T.; Olsen, T.; Pastewka, L.; Peterson, A.; Rostgaard, C.; Schiøtz, J.; Schütt, O.; Strange, M.; Thygesen, K. S.; Vegge, T.; Vilhelmsen, L.; Walter, M.; Zeng, Z.; Jacobsen, K. W. The Atomic Simulation Environment—a Python Library for Working with Atoms. *J. Phys. Condens. Matter,* **2017**, *29*, 273002.

(23) Paszke, A.; Gross, S.; Massa, F.; Lerer, A.; Bradbury, J.; Chanan, G.; Killeen, T.; Lin, Z.; Gimelshein, N.; Antiga, L., et al. Pytorch: An Imperative Style, High-Performance Deep Learning Library. *arXiv* **3 Dec 2019**, arXiv:1912.01703 (accessed 2025-10-04), DOI: 10.48550/arXiv.1912.01703.

(24) Kingma, D. P. Adam: A Method for Stochastic Optimization. *arXiv* **2014**, arXiv:1412.6980.

(25) Plimpton, S. Fast Parallel Algorithms for Short-Range Molecular Dynamics. *J. Comput. Phys.* **1995**, *117*, 1–19, DOI: 10.1006/jcph.1995.1039.

(26) Thompson, A. P.; Aktulga, H. M.; Berger, R.; Bolintineanu, D. S.; Brown, W. M.; Crozier, P. S.; In 'T Veld, P. J.; Kohlmeyer, A.; Moore, S. G.; Nguyen, T. D.; Shan, R.; Stevens, M. J.; Tranchida, J.; Trott, C.; Plimpton, S. J. LAMMPS - a Flexible Simulation Tool for Particle-Based Materials Modeling at the Atomic, Meso, and Continuum Scales. *Comput. Phys. Commun.* **2022**, *271*, 108171, DOI: 10.1016/j.cpc.2021.108171.

(27) Edwards, H. C.; Trott, C. R.; Sunderland, D. Kokkos: Enabling Manycore Performance Portability through Polymorphic Memory Access Patterns. *J. Parallel Distrib. Comput.* **2014**, *74*, 3202–3216, DOI: https://doi.org/10.1016/j.jpdc.2014.07.003.

(28) Trott, C. R.; Lebrun-Grandié, D.; Arndt, D.; Ciesko, J.; Dang, V.; Ellingwood, N.; Gayatri, R.; Harvey, E.; Hollman, D. S.; Ibanez, D.; Liber, N.; Madsen, J.; Miles, J.; Poliakoff, D.; Powell, A.; Rajamanickam, S.; Simberg, M.; Sunderland, D.; Turcksin, B.; Wilke, J. Kokkos 3: Programming Model Extensions for the Exascale Era. *IEEE Trans. Parallel. Distrib. Syst.* **2022**, *33*, 805–817, DOI: 10.1109/TPDS.2021.3097283.

(29) Bartók, A. P.; Kondor, R.; Csányi, G. On Representing Chemical Environments. *Phys. Rev. B* **2013**, *87*, 184115, DOI: 10.1103/PhysRevB.87.184115.

(30) Himanen, L.; Jäger, M. O. J.; Morooka, E. V.; Federici Canova, F.; Ranawat, Y. S.; Gao, D. Z.; Rinke, P.; Foster, A. S. DScribe: Library of Descriptors for Machine Learning in Materials Science. *Comput. Phys. Commun.* **2020**, *247*, 106949, DOI: 10.1016/j.cpc.2019.106949.

(31) Laakso, J.; Himanen, L.; Homm, H.; Morooka, E. V.; Jäger, M. O.; Todorović, M.; Rinke, P. Updates to the DScribe Library: New Descriptors and Derivatives. *J. Chem. Phys.* **2023**, *158*.

(32) Wold, S.; Esbensen, K.; Geladi, P. Principal Component Analysis. *Chemom. Intell. Lab. Syst.* **1987**, *2*, 37–52, DOI: 10.1016/0169-7439(87)80084-9.

(33) Pedregosa, F.; Varoquaux, G.; Gramfort, A.; Michel, V.; Thirion, B.; Grisel, O.; Blondel, M.; Prettenhofer, P.; Weiss, R.; Dubourg, V.; Vanderplas, J.; Passos, A.; Cournapeau, D.; Brucher, M.; Perrot, M.; Duchesnay, E. Scikit-Learn: Machine Learning in Python. *J. Mach. Learn. Res.* **2011**, *12*, 2825–2830.

(34) Maaten, L. v. d.; Hinton, G. Visualizing Data Using t-SNE. *Journal of machine learning research* **2008**, *9*, 2579–2605.

(35) Barnes, J.; Hut, P. A Hierarchical O(N log N) Force-Calculation Algorithm. *Nature* **1986**, *324*, 446–449, DOI: 10.1038/324446a0.

(36) Kullback, S.; Leibler, R. A. On Information and Sufficiency. *Ann. Math. Stat.* **1951**, *22*, 79–86.

(37) Stukowski, A. Visualization and Analysis of Atomistic Simulation Data with OVITO–the Open Visualization Tool. *Model. Simul. Mater. Sci. Eng.* **2009**, *18*, 015012, DOI: 10.1088/0965-0393/18/1/015012.

(38) Savitzky, A.; Golay, M. J. E. Smoothing and Differentiation of Data by Simplified Least Squares Procedures. *Anal. Chem.* **1964**, *36*, 1627–1639, DOI: 10.1021/ac60214a047.

(39) Ahlrichs, R.; Bär, M.; Häser, M.; Horn, H.; Kölmel, C. Electronic Structure Calculations on Workstation Computers: The Program System Turbomole. *Chem. Phys. Lett.* **1989**, *162*, 165–169, DOI: 10.1016/0009-2614(89)85118-8.

(40) Balasubramani, S. G.; Chen, G. P.; Coriani, S.; Diedenhofen, M.; Frank, M. S.; Franzke, Y. J.; Furche, F.; Grotjahn, R.; Harding, M. E.; Hättig, C.; Hellweg, A.; Helmich-Paris, B.; Holzer, C.; Huniar, U.; Kaupp, M.; Marefat Khah, A.; Karbalaei Khani, S.; Müller, T.; Mack, F.; Nguyen, B. D.; Parker, S. M.; Perlt, E.; Rappoport, D.; Reiter, K.; Roy, S.; Rückert, M.; Schmitz, G.; Sierka, M.; Tapavicza, E.; Tew, D. P.; van Wüllen, C.; Voora, V. K.; Weigend, F.; Wodyński, A.; Yu, J. M. TURBOMOLE: Modular Program Suite for Ab Initio Quantum-Chemical and Condensed-Matter Simulations. *J. Chem. Phys.* **2020**, *152*, 184107, DOI: 10.1063/5.0004635.

(41) TURBOMOLE GmbH TURBOMOLE V7.6 2021, http://www.turbomole.com, A Development of University of Karlsruhe and Forschungszentrum Karlsruhe GmbH, 1989-2007, TURBOMOLE GmbH, Since 2007, 2021.

(42) Franzke, Y. J.; Holzer, C.; Andersen, J. H.; Begušić, T.; Bruder, F.; Coriani, S.; Della Sala, F.; Fabiano, E.; Fedotov, D. A.; Fürst, S.; Gillhuber, S.; Grotjahn, R.; Kaupp, M.; Kehry, M.; Krstić, M.; Mack, F.; Majumdar, S.; Nguyen, B. D.; Parker, S. M.; Pauly, F.; Pausch, A.; Perlt, E.; Phun, G. S.; Rajabi, A.; Rappoport, D.; Samal, B.; Schrader, T.; Sharma, M.; Tapavicza, E.; Treß, R. S.; Voora, V.; Wodyński, A.; Yu, J. M.; Zerulla, B.; Furche, F.; Hättig, C.; Sierka, M.; Tew, D. P.; Weigend, F. TURBOMOLE: Today and Tomorrow. *J. Chem. Theory Comput.* **2023**, *19*, 6859–6890, DOI: 10.1021/acs.jctc.3c00347.

(43) Franzke, Y. J.; Weigend, F. NMR Shielding Tensors and Chemical Shifts in Scalar-Relativistic Local Exact Two-Component Theory. *J. Chem. Theory Comput.* **2019**, *15*, 1028–1043, DOI: 10.1021/acs.jctc.8b01084.

(44) J. Franzke, Y.; Treß, R.; M. Pazdera, T.; Weigend, F. Error-Consistent Segmented Contracted All-Electron Relativistic Basis Sets of Double- and Triple-Zeta Quality for NMR Shielding Constants. *Phys. Chem. Chem. Phys.* **2019**, *21*, 16658–16664, DOI: 10.1039/C9CP02382H.

(45) Pollak, P.; Weigend, F. Segmented Contracted Error-Consistent Basis Sets of Double- and Triple-Valence Quality for One- and Two-Component Relativistic All-Electron Calculations. *J. Chem. Theory Comput.* **2017**, *13*, 3696–3705, DOI: 10.1021/acs.jctc.7b00593.

(46) Becke, A. D. Density-Functional Exchange-Energy Approximation with Correct Asymptotic Behavior. *Phys. Rev. A* **1988**, *38*, 3098–3100, DOI: 10.1103/PhysRevA.38.3098.

(47) Lee, C.; Yang, W.; Parr, R. G. Development of the Colle-Salvetti Correlation-Energy Formula Into a Functional of the Electron Density. *Phys. Rev. B* **1988**, *37*, 785–789, DOI: 10.1103/PhysRevB.37.785.

(48) Becke, A. D. A New Mixing of Hartree-Fock and Local Density-Functional Theories. *J. Chem. Phys.* **1993**, *98*, 1372–1377, DOI: 10.1063/1.464304.

(49) Venetos, M. C.; Wen, M.; Persson, K. A. Machine Learning Full NMR Chemical Shift Tensors of Silicon Oxides with Equivariant Graph Neural Networks. *J. Phys. Chem. A* **2023**, *127*, 2388–2398, DOI: 10.1021/acs.jpca.2c07530.

(50) Wen, M.; Horton, M. K.; Munro, J. M.; Huck, P.; Persson, K. A. An Equivariant Graph Neural Network for the Elasticity Tensors of all Seven Crystal Systems. *Digit. Discov.* **2024**, *3*, 869–882, DOI: 10.1039/D3DD00233K.

(51) Harris, C. R.; Millman, K. J.; van der Walt, S. J.; Gommers, R.; Virtanen, P.; Cournapeau, D.; Wieser, E.; Taylor, J.; Berg, S.; Smith, N. J.; Kern, R.; Picus, M.; Hoyer, S.; van Kerkwijk, M. H.; Brett, M.; Haldane, A.; del Río, J. F.; Wiebe, M.; Peterson, P.; Gérard-Marchant, P.; Sheppard, K.; Reddy, T.; Weckesser, W.; Abbasi, H.; Gohlke, C.; Oliphant, T. E. Array Programming with NumPy. *Nature* **2020**, *585*, 357–362, DOI: 10.1038/s41586-020-2649-2.

(52) Hunter, J. D. Matplotlib: A 2D Graphics Environment. *Comput. Sci. Eng.* **2007**, *9*, 90–95, DOI: 10.1109/MCSE.2007.55.

(53) Waskom, M. L. Seaborn: Statistical Data Visualization. *J. Open Source Softw.* **2021**, *6*, 3021, DOI: 10.21105/joss.03021.

(54) Humphrey, W.; Dalke, A.; Schulten, K. VMD – Visual Molecular Dynamics. *J. Mol. Graph.* **1996**, *14*, 33–38.

(55) Stone, J. *An Efficient Library for Parallel Ray Tracing and Animation*, MA thesis, Computer Science Department, University of Missouri-Rolla, 1998.
